# Bazedoxifene Targets gp130 Signaling to Suppress Fibroblast Activation in Pathological Cutaneous Fibrosis: Insights from Spatial Transcriptomics and scRNA-seq Data

**DOI:** 10.7150/ijbs.137778

**Published:** 2026-08-24

**Authors:** Zixin Wang, Hanrui Zhang, Liying Tu, Jingjing He, Rong Wang, Yinghong Su, Siwei Tang, Qingfeng Li, Wenzheng Xia, Yashan Gao, Yixuan Zhao, Xin Huang, Tao Zan

**Affiliations:** Department of Plastic and Reconstructive Surgery, Shanghai Ninth People's Hospital, Shanghai Jiao Tong University School of Medicine, Shanghai, China

**Keywords:** bazedoxifene, cutaneous fibrosis, keloid, gp130, spatial transcriptomics

## Abstract

Pathological cutaneous fibrosis, exemplified by hypertrophic scarring and keloid formation, imposes substantial disease burden through persistent pruritus, pain and contracture-induced functional impairment. While the antifibrotic role of gp130 signaling inhibition has been established in visceral organ fibrosis, its therapeutic efficacy in pathological cutaneous fibrosis remains unexplored. Herein, we investigated the effectiveness of gp130 inhibition on cutaneous fibrosis by leveraging the application of Bazedoxifene, a clinically approved selective estrogen receptor modulator recently identified as a gp130 signaling inhibitor. We demonstrate that gp130 signaling is aberrantly activated in human keloid tissues and fibroblasts. *In vitro,* Bazedoxifene suppressed the fibroblast activation of disease-derived fibroblasts, featured by attenuated cell migration, myofibroblast differentiation and ECM production. *In vivo*, Bazedoxifene ameliorated dermal fibrosis and ECM deposition in both bleomycin-induced cutaneous fibrosis and keloid xenograft models. Notably, spatial transcriptomic analysis revealed reduced myofibroblast differentiation within fibrotic regions, providing tissue-level evidence for inhibition of fibroblast activation. Transcriptomic analysis revealed suppression of multiple fibrosis-associated signaling pathways following Bazedoxifene treatment, including the PI3K-AKT-mTOR, JAK-STAT, and MAPK pathways, with the PI3K-AKT-mTOR pathway showing the most prominent inhibition. Collectively, these findings establish Bazedoxifene repurposing as a promising therapeutic strategy for cutaneous fibrosis.

## Introduction

Pathological cutaneous fibrosis, exemplified by hypertrophic scarring and keloid formation, imposes substantial disease burden through persistent pruritus, chronic pain, contracture-induced functional impairment, which is driven by extracellular matrix (ECM) deposition 1. Current management strategies-including surgical excision with adjunctive radiotherapy, intralesional corticosteroid injections, and silicone-based pressure therapy-remain empirical and suboptimal, plagued by limited efficacy, high recurrence rates, and significant adverse effects 1, 2. This therapeutic void stems from persistent gaps in understanding the cellular and signaling mechanisms driving fibroblast dysregulation, underscoring the urgent unmet need for mechanism-based interventions.

At the cellular level, pathological fibrosis is characterized by persistent fibroblast activation and excessive ECM accumulation 3, 4. Activated fibroblasts exhibit enhanced migratory capacity, increased ECM production, and differentiation into contractile myofibroblasts, making fibroblast activation a central event in pathological tissue remodeling 4, 5. Among the multiple signaling pathways involved, transforming growth factor-β1 (TGF-β1) is widely recognized as a key regulator 6. However, its broad physiological roles in tissue homeostasis and immune regulation limit the feasibility of direct therapeutic targeting 7, 8.

Glycoprotein-130 (gp130) has emerged as a critical signal-transducing receptor that integrates cytokine inputs and regulates multiple biological processes, including inflammation, tissue repair, and fibrosis 9, 10. As the shared signal-transducing subunit of the interleukin-6 (IL-6) cytokine family, gp130 is recruited following ligand binding to specific α-receptor chains, leading to the formation of a ligand-receptor complex that assembles into a higher-order, signaling-competent structure 10. This structural rearrangement enables gp130 dimerization and initiates intracellular signal transduction 11-13. Accumulating evidence has highlighted the importance of gp130-dependent signaling in visceral organ fibrosis. Among gp130-associated cytokines, interleukin-11 (IL-11) has been identified as a key mediator of TGF-β1-driven fibrosis, with strong pathogenic roles demonstrated in cardiovascular 14, 15, pulmonary 16-18, and renal 19 fibrosis. In addition, several other gp130-dependent cytokines, including oncostatin M (OSM), leukemia inhibitory factor (LIF), and cardiotrophin-1 (CT-1), have also been implicated in regulating fibroblast activation, inflammatory responses, and ECM remodeling in a context-dependent manner 20-24. Therefore, as the shared signal-transducing subunit of the IL-6 cytokine family, targeting gp130 may modulate a broader fibroinflammatory signaling network than targeting a single cytokine pathway and thus represents an attractive therapeutic strategy for fibrotic diseases.

Despite the predominant role of gp130 in fibrosis, therapeutic strategies of targeting gp130 in skin fibrosis remain limited 25-27. Biologic approaches targeting gp130, such as the soluble gp130Fc fusion protein, have entered clinical and translational evaluation in inflammatory and fibrotic diseases 28-30. However, these agents selectively inhibit IL-6 trans-signaling by neutralizing the IL-6/sIL-6R complex, rather than directly blocking membrane-bound gp130 or broadly suppressing gp130-mediated signaling pathways 31. In contrast, several small-molecule compounds, including SC-144, have been reported to interfere with gp130-mediated signaling and have been primarily investigated in cancer 32, inflammation 33, and cardiopulmonary disease 34-36. Nevertheless, these agents remain at the preclinical stage and are limited by suboptimal specificity and lack of clinical translation 36.

Bazedoxifene is an FDA-approved selective estrogen receptor modulator (SERM) that has been widely used for the prevention and treatment of postmenopausal osteoporosis, possessing a well-established clinical safety profile. In 2014, Bazedoxifene was identified as a gp130 inhibitor 37. Bazedoxifene disrupts IL-6 family cytokine signaling by binding to gp130 and interfering with ligand-induced receptor complex formation, thereby preventing gp130 dimerization and activation of downstream pathways 38. In pancreatic cancer models, Bazedoxifene has been reported to suppress IL-6/gp130-mediated STAT3 activation and tumor cell proliferation 38. In addition, Bazedoxifene ameliorated cardiac remodeling through inhibition of IL-6/gp130 signaling 39. Its anti-fibrotic effects have also been reported in systemic sclerosis, a systemic autoimmune disease characterized by widespread sclerosis and immune dysregulation where Bazedoxifene reduced fibroblast activation 40. Collectively, these pharmacological characteristics and preclinical findings provide a strong rationale for evaluating Bazedoxifene as a therapeutic strategy for pathological cutaneous fibrosis. However, the role of Bazedoxifene in pathological cutaneous fibrosis, particularly in localized fibrotic lesions such as hypertrophic scars and keloids, has not been systematically investigated.

Given the central role of gp130 signaling in fibroblast activation and fibrotic remodeling, we hypothesized that Bazedoxifene may exert anti-fibrotic effects in cutaneous fibrosis by disrupting gp130-mediated signaling. Based on the profibrotic effect of IL-11 and IL-6 14, 41, 42, these two cytokines were selected as representative ligands of the gp130 signaling axis for further investigation. This study systematically assessed the effects of Bazedoxifene on fibroblast migration, myofibroblast differentiation and ECM deposition, and further explored the underlying molecular mechanisms and transcriptional programs associated with fibroblast activation. By integrating cellular, transcriptomic, and* in vivo* models, this study seeks to define the role of gp130-driven signaling in cutaneous fibrosis and to evaluate the potential of Bazedoxifene for clinical repositioning in the treatment of cutaneous fibrosis.

## Materials and Methods

### scRNA-seq data integration and preprocessing

To investigate fibroblast heterogeneity and gp130-related signaling in cutaneous fibrosis, publicly available single-cell RNA sequencing (scRNA-seq) datasets were collected from the Gene Expression Omnibus (GEO) and the Genome Sequence Archive (GSA), including keloid, hypertrophic scar (HTS), normal scar, and normal skin samples (GSE156326, GSE181297, GSE181316, GSE266334, GSE163973, and HRA000425). In total, 34 samples were included for analysis. All datasets were processed using a unified analysis pipeline to ensure comparability. Gene expression matrices were imported into R (v4.5.2) and analyzed using the Seurat package (v5.3.1). Cells with low quality (fewer than 200 detected genes) were excluded, and genes expressed in fewer than three cells were removed. Cells with a high proportion of mitochondrial gene expression were also filtered out based on standard quality control criteria.

After quality control, data were normalized and 3,000 highly variable genes were identified for downstream analysis. Data integration across datasets was performed using Seurat (FindIntegrationAnchors and IntegrateData functions) to correct for batch effects. Principal component analysis (PCA) was conducted, and the top 30 principal components were used for clustering and dimensionality reduction. Uniform Manifold Approximation and Projection (UMAP) was applied for visualization of cell distributions. Fibroblast populations were extracted based on canonical marker genes and further subclustered. Cell identities were assigned based on established marker genes, and fibroblasts were classified into four subpopulations: mesenchymal fibroblasts, pro-inflammatory fibroblasts, secretory-papillary fibroblasts, and secretory-reticular fibroblasts 43. Gene expression analysis of *IL6*, *IL6R*, *IL11*, and *IL11RA* was performed across fibroblast subpopulations and tissue types. Gene expression distributions were visualized using violin plots, and differential expression patterns were compared between keloid, HTS, and control samples.

### Cells, clinical samples, and ethics statement

Keloid, HTS and normal skin samples were obtained from patients who underwent surgery at the Department of Plastic and Reconstructive Surgery at Shanghai Ninth People's Hospital, Shanghai Jiao Tong University School of Medicine. All participants provided written informed consent, and human tissue collection was conducted in accordance with protocols approved by the Ethics Committee of Shanghai Ninth People's Hospital (patient information is summarized in Table S1). The ethics permit number for the use of clinical samples collected during surgery was 2018-129-T107.

### Cell culture and treatment

Isolation and culture of human keloid and normal skin fibroblasts were performed as previously described 44. Specifically, specimens obtained during surgery were cut into 5 mm × 5 mm pieces and soaked in 0.3% dispase II (3 g/mL; Gibco, 17105041) at 4 °C for 12 h. Then, the epidermis was removed, and the dermis was minced and incubated in collagenase NB4 (3 mg/mL; Nordmark, S1745401) at 37 °C for 4 h to isolate dermal fibroblasts. Fibroblasts were cultured in DMEM (Gibco, USA) supplemented with 10% fetal bovine serum (FBS; Gibco, USA) and 1% penicillin-streptomycin (Gibco, USA) and then incubated at 37 °C in a humidified atmosphere with 5% CO2. After approximately 4-5 days, the isolated dermal fibroblasts began to adhere, and after another 48 h of culture, the density reached approximately 90%. At this time point, fibroblasts were passaged at a 1:3 ratio. Afterward, the fibroblasts were cultured under the conditions mentioned above, and passages were performed every 72 h for a maximum of six passages.

Cells were seeded in plates of appropriate formats and serum-starved for 12 h prior to treatment. Cells were then treated with Bazedoxifene, IL-11, IL-6 or Bazedoxifene in combination with IL-11 or IL-6 in DMEM without FBS for 24-48 h as indicated. Vehicle-treated cells served as controls, and the final concentration of DMSO was kept consistent across all groups.

### Immunofluorescence (IF) and western blotting (WB) analyses

WB analysis and IF staining were performed according to the protocol described in our previous study 25. The antibodies used for WB and IF are listed in Table S3. The original WB images are available in Original Data. Densitometric quantification of WB and quantification of IF intensity were determined using ImageJ software. For co-localization analysis, fluorescence intensity profiles were generated using line-scan analysis in ImageJ software. A line was drawn across the region of interest, and fluorescence intensities of each channel along the selected path were extracted to assess spatial co-distribution of signals.

### Reagents and drug preparation

Bazedoxifene acetate (TargetMol, T2544, USA) was dissolved in dimethyl sulfoxide (DMSO) to prepare a stock solution according to the manufacturer's instructions. The stock solution was stored at -20 °C and diluted to the indicated working concentrations in DMEM without FBS prior to use. The final concentration of DMSO was kept consistent across all experimental groups.

Recombinant human IL-11 (MCE, HY-P7031) and IL-6 (MCE, HY-P7044) were reconstituted in double-distilled water (ddH2O) according to the manufacturer's instructions. Cytokines were aliquoted and stored at -80 °C, and diluted to the indicated concentrations in culture medium before use.

### Cell Viability Assays

CCK-8 colorimetric assays were used to assess cell viability. Normal skin fibroblasts and keloid fibroblasts were seeded into 96-well plates (Corning, USA) at a density of 3000-5000 cells per well in 100 μL of complete medium and allowed to adhere overnight. Cells were then incubated in DMEM without FBS containing the indicated concentrations of Bazedoxifene for 48 h. 3 h prior to detection, 10 μL of CCK-8 solution (Dojindo, Japan) was added to each well, followed by incubation at 37 °C. Absorbance at 450 nm was measured using a microplate reader (ELX800, BioTek, USA). Cell viability was normalized to the vehicle-treated control group and expressed as a percentage of growth.

### Wound healing assay

Cell migration was evaluated using a wound healing assay. Normal skin fibroblasts and keloid fibroblasts were seeded into 6-well plates and cultured in complete medium until reaching approximately 90-100% confluence. A linear scratch was created across the cell monolayer using a sterile 200 μL pipette tip. Detached cells were gently removed by washing with PBS, and cells were then incubated in DMEM without FBS. The wound area was measured using ImageJ software, and the percentage of wound closure was calculated as follows: migrated area (%) = (initial wound area - remaining wound area) / initial wound area × 100%.

### Transwell assay

A 24-well Transwell system with polycarbonate filters (8 μm pores; Corning, USA) was used. The upper chamber contained 1 × 10^5^ cells suspended in 200 μL of DMEM without FBS, and the lower chamber contained 500 μL of DMEM with 20% FBS. Cells in the upper chamber were treated with the indicated conditions, including vehicle, Bazedoxifene alone, cytokine stimulation (IL-11 or IL-6), or cytokine stimulation in combination with Bazedoxifene. After 24 h or 48 h of incubation at 37 °C, the cells were stained with 0.1% crystal violet. The cells in the upper chamber were removed, and the migrated cells were photographed and counted by Image J software.

### RNA isolation and quantitative reverse-transcription PCR (qRT‒PCR)

Total RNA was extracted using TRIzol reagent (Solarbio, China). Complementary DNA was synthesized from 2000 ng of RNA with PrimeScript RT Master Mix (TaKaRa, Japan). PCR was performed on an ABI QuantStudio 6 Flex system using SYBR Premix (TaKaRa, Japan) according to the manufacturer's instructions. The sequences of the primers used are summarized in Table S2.

### RNA Interference

Knockdown of *IL6ST* expression in fibroblasts was achieved by transfection with two independent siRNAs synthesized by Genomeditech (Shanghai) Co., Ltd. The sequences of the siRNAs are listed in Table S4. Transfection of siRNAs was performed using Lipofectamine 3000 transfection reagent (Invitrogen, L3000008) according to the manufacturer's instructions. Knockdown efficiency was evaluated by qRT-PCR and Western blotting 48 h after transfection. siIL6ST-1, which exhibited higher knockdown efficiency, was selected for subsequent functional analyses. For functional assays, culture medium was replaced 24 h after siRNA transfection, followed by treatment with Bazedoxifene (5 μM) or vehicle for an additional 24 h. For IL-11 stimulation experiments, cells were treated with recombinant human IL-11 (20 ng/mL) in the presence or absence of Bazedoxifene for an additional 24 h after medium replacement.

### RNA-seq and data analysis

Total RNA was extracted from keloid-derived fibroblasts (KDFs) treated with Bazedoxifene (5 μM) or vehicle for 24 h (n = 3). mRNA was purified and used for library construction following the manufacturer's instructions (GenSeq, Inc., Shanghai, China). Libraries were sequenced on an Illumina platform using paired-end 150 bp reads. Raw reads were processed using fastp (v0.23.4) for quality control and adapter trimming. Clean reads were aligned to the reference genome using HISAT2 (v2.2.1), and gene-level raw counts were generated using featureCounts (v2.0.6). Raw count data were normalized and analyzed for differential expression using edgeR (v4.0.16). Genes with *P* < 0.05 and |log2(fold change)| ≥ 1 were considered differentially expressed.

Gene Ontology (GO) and Kyoto Encyclopedia of Genes and Genomes (KEGG) enrichment analyses were performed using DAVID. GO terms and KEGG pathways with adjusted *P* value < 0.05 were considered significant. Gene Set Enrichment Analysis (GSEA, v4.3.2) was performed using KEGG pathway gene sets obtained from the KEGG database, following standard procedures. Bubble plots were generated using Cytoscape (version 3.7.0), and read coverage tracks were visualized using Integrative Genomics Viewer (IGV). Downstream candidate genes and canonical signaling pathways were further analyzed using Ingenuity Pathway Analysis (IPA) via the Shanghai Jiao Tong University School of Medicine library.

### Animal maintenance and *in vivo* experiments

Male wildtype C57BL/6 mice and BALB/c nude mice (6-8 weeks old) were obtained from the Central Laboratory of Shanghai Ninth People's Hospital (Shanghai, China). All animal procedures were approved by the Ethics Committee of Shanghai Jiao Tong University School of Medicine and conducted in accordance with the Guide for the Care and Use of Laboratory Animals. The ethics permit number for the animal experiment was SH9H-2021-A178-1.

For the bleomycin-induced skin scarring model, mice were randomly divided into three groups (n = 6 per group): Sham, Bleo+DMSO, and Bleo+Baz. In the Bleo+DMSO and Bleo+Baz groups, 100 µL of bleomycin solution (1 U/mL; Sigma-Aldrich, B8416) was intradermally injected into the dorsal skin every other day for 30 days to induce fibrosis. Subsequently, mice in the Bleo+Baz group received intradermal injections of Bazedoxifene (100 µL, 5 mg/kg) at the same dorsal sites every other day, while mice in the Bleo+DMSO group received an equal volume of DMSO as vehicle control. Bleomycin administration was maintained throughout the treatment period. Mice in the Sham group received intradermal injections of an equal volume of PBS at the same dorsal sites on the same schedule.

For the patient-derived keloid xenograft model, fresh human keloid tissues were trimmed into uniform-sized grafts using a biopsy punch, with excess adipose tissue carefully removed. Each keloid specimen was then divided into two equal-sized grafts, weighed, and kept in cold PBS on ice prior to transplantation. Subcutaneous pockets were created in the bilateral dorsal axillary regions of BALB/c nude mice (n = 8), and the paired grafts from the same patient were implanted into the left and right sides of the same mouse. Beginning on postoperative day (POD) 3, intralesional injections were administered every 2 days until POD33. For each mouse, one graft was treated with DMSO as the vehicle control, while the contralateral graft received Bazedoxifene (5 mg/kg). This paired design allowed each mouse to serve as its own internal control. All mice were euthanized on POD33, and the xenografts were harvested, photographed, and weighed.

### Histological analysis

Tissues were fixed in 4% paraformaldehyde, dehydrated and then embedded in paraffin. Five-micrometer sections were sliced and stained with HE, Masson's Trichrome, and Picrosirius red staining kits following a standardized protocol. Sections were visualized with ECLIPSE Ni-E upright microscope (Nikon). Quantification of dermal thickness and collagen content was performed using ImageJ software. Quantitative analysis of overall ECM alignment was performed on picrosirius red-stained images under polarized light using the OrientationJ software package 45.

### Spatial transcriptomic analysis

Spatial transcriptomic data were generated using the DBiT-seq platform. Tissue sections were processed using a microfluidic barcoding strategy to encode spatial information of captured transcripts. Libraries were sequenced on an Illumina platform. Raw sequencing data were processed using the CeleScope pipeline for read alignment, gene expression quantification, and initial quality control. The resulting expression matrices were further filtered to remove low-quality spots based on standard quality control metrics. Spots with fewer than 200 or more than 7,500 detected genes, fewer than 200 or more than 20,000 UMI counts, or greater than 10% mitochondrial gene content were excluded from downstream analyses. Downstream analyses were performed using the Seurat workflow. Data normalization, identification of highly variable genes, scaling, and principal component analysis (PCA) were conducted following standard procedures. Unsupervised clustering was performed based on shared nearest neighbor (SNN) graph construction, and clusters were visualized using UMAP. Cell type annotation was performed based on canonical marker genes. Spatial mapping of cell types was visualized to assess tissue architecture across treatment groups. Myofibroblast differentiation scores were calculated based on gene sets associated with actin cytoskeleton organization and contractility 4, 27, derived from Gene Ontology Biological Process (GO-BP) terms. Scores were computed at the spot level to quantify the activation status across spatial regions.

### Statistical analysis

Statistical analyses were performed using GraphPad Prism 9. Data are presented as the mean ± standard deviation (SD). For relative expression analysis, the control group was set to 1 or 100% for normalization. Quantitative real-time PCR (qRT-PCR) data were analyzed using the comparative threshold cycle (ΔΔCt) method. Differences between two groups were analyzed using unpaired two-sided Student's t test. For paired data (e.g., keloid xenografts implanted in the same mouse), paired Student's t test was applied. Comparisons among multiple groups were performed using one-way analysis of variance (ANOVA), followed by multiple comparisons. A *P* value < 0.05 was considered statistically significant. Statistical significance is indicated as follows: ns, not significant; *P < 0.05*,* **P < 0.01*,* ***P < 0.001.

## Results

### gp130 Signaling is Aberrantly Activated in Pathological Scars

To comprehensively characterize the expression of gp130 signaling at single-cell resolution in human cutaneous fibrosis, we integrated six publicly available single-cell transcriptomic datasets from 34 samples including keloid, hypertrophic scar (HTS), normal scar, and normal skin to construct a unified human scar atlas (Figure 1A; datasets: GSE156326, GSE181297, GSE181316, GSE266334, GSE163973, and HRA000425). Based on established marker genes from previous studies, fibroblasts were further classified into four major subpopulations, including mesenchymal fibroblasts, pro-inflammatory fibroblasts, secretory-papillary fibroblasts, and secretory-reticular fibroblasts 43 (Figure 1B). Notably, mesenchymal fibroblasts were markedly expanded in pathological fibrotic conditions, particularly in keloid and HTS tissues, implicating their pivotal role in pathological tissue remodeling (Figure 1B). Given their close association with ECM production and myofibroblast differentiation, mesenchymal fibroblasts were considered a key pathogenic subpopulation 43.

Given the established roles of IL-11 and IL-6 in fibrotic remodeling, these cytokines and their cognate receptors were selected for focused investigation. Expression profiling revealed that *IL11*, *IL6*, *IL11RA*, and *IL6R* were all expressed in pathogenic mesenchymal fibroblasts, with *IL11* exhibiting significantly higher expression relative to other fibroblast subpopulations (Figure 1C). Notably, when focusing on mesenchymal fibroblasts, the expression levels of *IL11*, *IL6*, *IL11RA*, and *IL6R* were all elevated in keloid and HTS compared with normal skin or normal scars, with particularly pronounced upregulation in keloid tissues (Figure 1D). These findings suggest enhanced activation of gp130-dependent cytokine signaling in disease-associated fibroblasts.

Keloids, a representative manifestation of pathological skin fibrosis, were selected as a model to further validate the status of gp130 signaling in human tissue. We then analyzed the expression of key receptor components in specimens obtained from surgically excised keloids and normal skin, and clinical characteristics of the patients are summarized in Table S1. Immunofluorescence (IF) staining demonstrated strong enrichment of IL-11Rα and IL-6Rα signals within the fibrotic dermal regions of keloid lesions (Figure 1E). Quantitative analysis confirmed significantly elevated receptor expression in keloid compared with normal skin (Figure 1F, G). Co-localization analysis further demonstrated spatial overlap with α-SMA-positive fibrotic regions, supporting their association with activated fibroblasts (Figure 1H, I). Notably, similar increases in IL-11Rα and IL-6Rα expression were also observed in hypertrophic scar tissues compared with normal skin (Figure S1A-C), further supporting activation of gp130-associated signaling in pathological cutaneous fibrosis.

We next investigated whether these alterations were inherent to fibroblasts derived from keloids and normal skin tissues. IF staining revealed markedly higher expression of IL-11Rα, IL-6Rα, and gp130 in keloid-derived fibroblasts (KDFs) compared with normal dermal fibroblasts (NDFs) (Figure 1J, K). Western blotting further confirmed significantly increased protein levels of these receptors in keloid fibroblasts (Figure S2A, B). Consistently, ELISA assays demonstrated elevated secretion of IL-6 and IL-11 in culture supernatants of keloid fibroblasts (Figure 1L), suggesting enhanced cytokine signaling activity. Together, these findings indicate the aberrant activation of the gp130 signaling at both tissue and cellular levels in pathological fibrosis.

### Bazedoxifene Inhibits Fibroblast Migration, Myofibroblast Differentiation and ECM Production in Keloid-derived Fibroblasts

To evaluate the biological effects of Bazedoxifene on keloid-derived fibroblasts (KDFs), we first assessed its cytotoxic profile. Primary fibroblasts isolated from keloid tissues and normal skin were treated with escalating concentrations of Bazedoxifene for 48 hours (Figure 2A). Cell Counting Kit-8 (CCK-8) assays demonstrated that, although KDFs exhibited heightened sensitivity to Bazedoxifene at elevated concentrations, viability in both cell types remained above 90% at concentrations up to 5 μM. These findings establish that Bazedoxifene concentrations ≤5 μM do not induce significant cytotoxicity, thereby defining a therapeutically relevant dosing range for subsequent functional studies. EdU incorporation and flow cytometry analyses further confirmed that Bazedoxifene at these concentrations did not significantly affect fibroblast proliferation, apoptosis or cell cycle (Figure S3A-C).

We next examined whether Bazedoxifene affects KDF migratory behavior. Scratch-wound assays revealed that Bazedoxifene markedly reduced wound closure in keloid fibroblasts in a dose-dependent manner over 24 and 48 h (Figure 2C, D). Consistently, transwell migration assays showed significantly fewer migrating cells following Bazedoxifene treatment compared with vehicle controls (Figure 2E, F), confirming that Bazedoxifene suppresses fibroblast motility.

In addition, Bazedoxifene markedly attenuated myofibroblast differentiation and ECM production in KDFs. Quantitative reverse transcription-PCR (qRT-PCR) showed that Bazedoxifene significantly reduced the expression of *ACTA2*, the canonical marker of myofibroblast differentiation (Figure 2G). Consistent with this finding, protein analysis confirmed a dose-dependent decrease in α-SMA levels following Bazedoxifene treatment (Figure 2H, I). Beyond its effect on myofibroblast differentiation, Bazedoxifene also significantly downregulated the expression of major ECM genes, including *COL1A1*, *COL3A1*, and *FN1*, at both mRNA and protein levels (Figure 2G-I). IF staining further confirmed that Bazedoxifene decreased the abundance of α-SMA-positive cells and reduced deposition of ECM proteins in keloid fibroblasts (Figure 2J-K). Together, these findings demonstrate that Bazedoxifene not only inhibits fibroblast motility but also suppresses both myofibroblast differentiation and ECM production, two key pathological hallmarks of fibroblast activation in keloids.

### Bazedoxifene Attenuates IL-11/IL-6-induced Fibroblast Migration, Myofibroblast Differentiation and ECM Production

To determine whether Bazedoxifene can counteract cytokine-induced profibrotic responses, we next examined its effects on normal-skin-derived fibroblasts under IL-11 or IL-6 stimulations. qRT-PCR analysis showed that both IL-11 and IL-6 stimulation upregulated the expression of fibrotic genes, including *ACTA2*, *COL1A1*, *COL3A1*, and *FN1* in NDFs (Figure S4A-D). Bazedoxifene significantly attenuated the expression of these genes in a dose-dependent manner after IL-11 (20 ng/mL) stimulation (Figure 3A-D). In contrast, IL-6 (40 ng/mL) stimulation induced milder transcriptional changes, which were likewise reversed by Bazedoxifene. Consistent with this observation, our integrated scRNA-seq analysis revealed that *IL11* and *IL11RA* were preferentially expressed in fibroblasts, whereas *IL6* and *IL6R* were more broadly distributed across stromal, epithelial, and immune cell populations (Figure S5), supporting the notion that IL-11 may act more specifically on the functional regulation of fibroblasts, whereas the IL-6 may have a broader function as an immunomodulatory cytokine as previously reported 41, 42, 46. Western Blotting demonstrated that IL-11 stimulation enhanced the levels of profibrotic proteins, whereas Bazedoxifene markedly reduced their expression (Figure 3E, F). Similar trends were observed under IL-6 stimulation, further confirming that Bazedoxifene suppresses cytokine-driven myofibroblast differentiation and ECM production (Figure 3G).

We next assessed whether Bazedoxifene affects fibroblast motility in cytokine-activated conditions. Scratch-wound assays revealed that IL-11 and IL-6 both enhanced wound closures compared with vehicle controls, while Bazedoxifene treatment significantly impaired this cytokine-induced migratory response (Figure 3H-I). Transwell assays further confirmed a marked reduction in migrated cells following Bazedoxifene treatment (Figure 3J-K).

Together, these findings demonstrate that Bazedoxifene effectively attenuates IL-11- and IL-6-induced myofibroblast differentiation, ECM production, and migratory behavior, indicating that Bazedoxifene interferes with cytokine-driven fibrotic programming in fibroblasts.

### Bazedoxifene Regulates Fibroblast Activation Through gp130 Signaling

To further determine whether the antifibrotic effects of Bazedoxifene are dependent on gp130 signaling, *IL6ST* (gp130) was initially silenced using siRNAs. The one that most efficiently reduced* IL6ST* expression at both the mRNA and protein levels was selected for subsequent functional analyses (Figure 4A-C). Genetic silencing of *IL6ST* significantly suppressed the expression of *ACTA2*, *COL1A1*, *COL3A1* and *FN1* in KDFs. Following Bazedoxifene treatment, no significant additional inhibition of these profibrotic markers was observed, suggesting that the antifibrotic effects of Bazedoxifene are mainly mediated through gp130 signaling (Figure 4D-F).

Furthermore, IL-11-induced myofibroblast differentiation and ECM production were largely abolished by *IL6ST* knockdown. Following Bazedoxifene treatment, no significant additional changes were observed in the examined profibrotic markers at either the mRNA or protein levels in IL6ST-silenced NDFs (Figure 4G-O). Taken together, these findings provide genetic evidence that the regulatory effects of Bazedoxifene on fibroblast activation are largely mediated through gp130 signaling.

### Bazedoxifene Attenuates Dermal Fibrosis *In vivo*

To investigate the anti-fibrotic effect of Bazedoxifene *in vivo*, we employed a bleomycin-induced murine skin fibrosis model 47 and a patient-derived keloid xenograft model 48 to mimic human pathological scars (Figure 5A, F). In the bleomycin model, Bazedoxifene significantly reduced dermal thickness and collagen deposition in HE and Masson trichrome staining (Figure 5B). Histological analysis further revealed markedly improved collagen organization, decreased collagen I/III ratio and reduced fibronectin deposition following Bazedoxifene treatment (Figure 5C, D). The orientation of ECM fibers was quantitatively evaluated by OrientationJ algorithm 45. Bazedoxifene-treated scars exhibited a looser and more dispersed pattern, compared to the concentrated and aligned collagen architecture observed in vehicle controls (Figure 5C, E and Figure S6A). Consistent with these histological findings, the expression of fibrotic markers was significantly reduced at both mRNA and protein levels (Figure S7A, B). In keloid xenograft model, the gross appearance and weight of keloid xenografts were significantly reduced compared with vehicle controls (Figure 5G). Consistent with the observations in the bleomycin model, Bazedoxifene treatment reduced collagen content, decreased the collagen I/III ratio, improved collagen fiber organization, and diminished fibronectin deposition in xenograft tissues (Figure 5H-J, Figure S6B and Figure S7C, D).

### Bazedoxifene Suppresses Myofibroblast Differentiation *In vivo*

To further characterize the anti-fibrotic effects of Bazedoxifene at the spatial and transcriptional levels, we performed spatial transcriptomic analysis on skin tissues derived from the bleomycin-induced fibrosis model (Figure 6A; GEO: GSE326315). Unsupervised clustering and cell-type annotation revealed distinct stromal and epithelial populations, including fibroblasts, keratinocytes, hair follicle cells, Schwann cells, and vascular-associated cells (Figure 6B-D). Among these, fibroblasts exhibited prominent enrichment of ECM-related genes, including *Col1a1*, *Col3a1*, and *Fn1*, consistent with their central role in fibrotic remodeling (Figure 6C).

To quantitatively assess myofibroblast differentiation states, we established a myofibroblast differentiation score based on gene sets related to actin cytoskeleton organization and contractility 4, 27, derived from Gene Ontology Biological Process (GO-BP) annotations. Spatial mapping demonstrated that fibrotic regions in vehicle (DMSO)-treated tissues exhibited markedly higher activation scores, whereas Bazedoxifene treatment substantially reduced this activation signal across the tissue landscape (Figure 6E). Quantitative analysis further confirmed a significant decrease in myofibroblast differentiation scores following Bazedoxifene treatment (Figure 6F), indicating effective suppression of fibroblast activation *in vivo*.

Consistent with these spatial transcriptomic findings, immunofluorescence staining revealed a reduction in α-SMA-positive myofibroblasts in both the bleomycin-induced fibrosis model and the patient-derived keloid xenograft model upon Bazedoxifene treatment (Figure 6G). These results collectively demonstrate that Bazedoxifene attenuates myofibroblast differentiation *in vivo*, thereby contributing to the inhibition of pathological ECM remodeling.

### Bulk RNA-seq Reveals Suppression of Fibroblast Activation-related Signaling by Bazedoxifene

To further elucidate the molecular mechanisms underlying the anti-fibrotic effects of Bazedoxifene, we performed a genome-wide transcriptome analysis (GEO: GSE325896) on KDFs treated with Bazedoxifene (5 μM) or vehicle (n = 3, Figure 7A). A distinct transcriptomic pattern was observed following Bazedoxifene treatment, with 1153 upregulated and 810 downregulated mRNAs, including representative genes highly relevant to fibrotic remodeling, such as *COL1A1*, *ACTA2*, and *CTGF,* together with upregulation of genes associated with reduced cytoskeletal contractility and ECM remodeling, including *ARHGAP5*, *RND3*, and* MMP12* (Figure 7B). Ingenuity Pathway Analysis (IPA) of canonical pathways and GO-BP enrichment revealed that differentially expressed genes were significantly associated with collagen biosynthesis, extracellular matrix organization, actin cytoskeleton regulation, and cell-substrate adhesion (Figure 7C, D). Together, these findings suggest that Bazedoxifene broadly suppresses key processes driving fibroblast activation, including ECM production, myofibroblast differentiation, and fibroblast migration, at the transcriptional level.

Gene set enrichment analysis (GSEA) further confirmed significant inhibition of pathways associated with these three profibrotic processes following Bazedoxifene treatment (Figure 7E, G, I). Consistently, heatmap visualization demonstrated a broad downregulation of profibrotic genes within these enriched gene sets. Inspection of RNA-seq coverage tracks further validated reduced transcriptional signals of representative fibrotic genes,* COL1A1*, *ACTA2*, and *NEDD9*, corresponding to ECM production, myofibroblast differentiation, and fibroblast migration, respectively, in Bazedoxifene-treated samples relative to vehicle controls (Figure 7F, H, J).

Collectively, these transcriptomic analyses indicate that Bazedoxifene suppresses the three key fibroblast activation-related processes in keloid fibroblasts, consistent with our *in vitro* and *in vivo* findings.

### Bazedoxifene Suppresses the PI3K-AKT-mTOR Pathway Downstream of the gp130 Axis

Previous studies have demonstrated that the IL-6/IL-11-gp130 signaling axis mediates profibrotic responses through several canonical downstream pathways, including JAK/STAT3, MAPK/ERK, and PI3K/AKT signaling 11, 12 (Figure S8A). To illustrate the molecular mechanisms of the anti-fibrotic effects of Bazedoxifene, we performed IPA-based network prediction analysis, which revealed a global predicted inhibition of gp130-related signaling networks after Bazedoxifene treatment, involving multiple downstream signaling nodes associated with STAT3, ERK, and PI3K pathways (Figure S8B). Furthermore, KEGG pathway enrichment analysis showed that among these pathways, PI3K/AKT signaling exhibited the most predominant enrichment (Figure 8A). GSEA further confirmed significant suppression of the PI3K/AKT pathway in Bazedoxifene-treated fibroblasts (Figure 8B). Consistent with the transcriptomic predictions, Bazedoxifene dose-dependently reduced the phosphorylation levels of key components of the PI3K-AKT-mTOR axis (Figure 8C, D), indicating suppression of PI3K-AKT signaling.

To determine whether this signaling inhibition occurs *in vivo*, IF staining was performed on normal murine tissue and fibrotic skin tissues from the bleomycin-induced fibrosis model. p-mTOR signals were predominantly localized in the cytoplasm of α-SMA-positive myofibroblasts within fibrotic lesions, whereas Bazedoxifene treatment markedly reduced p-mTOR activation in these cells (Figure 8E, F). Given that mTOR signaling primarily regulates cytoplasmic protein translation and ECM synthesis 49, 50, these findings suggest that Bazedoxifene suppresses the translational output of profibrotic signaling in activated fibroblasts.

In addition, other canonical gp130 downstream pathways, including STAT3 and ERK signaling, were also examined. GSEA revealed a decreasing trend in these pathways after Bazedoxifene treatment; however, these changes did not reach statistical significance (Figure S9A, B). Consistently, Bazedoxifene induced comparatively modest alterations in these signaling molecules at the protein level (Figure S9C). These molecules are typically enriched in the nucleus following activation and regulate fibrosis-related transcriptional programs through nuclear translocation 13, 51 (Figure S9D-F). Together, these findings suggest that Bazedoxifene simultaneously attenuates both cytoplasmic translational signaling and nuclear transcriptional regulation, with the PI3K-AKT-mTOR axis representing the dominant pathway affected in keloid fibroblasts.

## Discussion

Pathological scarring is characterized by persistent fibroblast activation and excessive ECM deposition, yet the precise molecular mechanisms governing this process remain incompletely understood. In this study, we demonstrate aberrant activation of gp130 signaling in pathological cutaneous fibrosis and show that Bazedoxifene suppresses fibroblast activation both *in vitro* and *in vivo*, primarily through inhibition of the PI3K-AKT-mTOR pathway downstream of gp130 (Figure 8G).

Fibroblast activation represents a pivotal cellular event during fibrotic remodeling. Throughout this process, fibroblasts acquire contractile properties, enhanced migratory capacity, and increased ECM synthetic activity, ultimately culminating in persistent scar formation 52-54. Accordingly, elucidation of the signaling pathways that sustain fibroblast activation is essential for advancing our understanding of fibrotic pathogenesis and for developing targeted therapeutic strategies.

Although TGF-β1 is widely recognized as a central driver of fibrotic remodeling, its broad physiological functions limit the feasibility of direct therapeutic targeting 7, 8. Consequently, increasing attention has shifted toward identifying downstream mediators of TGF-β signaling as more specific therapeutic targets. Among these, members of the IL-6 cytokine family, particularly IL-11 and IL-6, have emerged as key executors of TGF-β-driven fibrotic responses. Upon TGF-β1 stimulation, fibroblasts markedly upregulate and secrete IL-11 and IL-6 14, which signal through gp130 to sustain fibroblast activation. IL-11 has been shown to form an autocrine signaling loop in fibroblasts that maintains their activated state 19. In agreement with these observations, we detected increased expression of gp130 receptor components in keloid tissues and fibroblasts together with elevated secretion of IL-6 and IL-11, suggesting that cytokine-driven gp130 signaling may contribute to maintaining fibroblast activation and the fibrotic microenvironment in pathological scars. Previous studies have established the involvement of gp130 signaling in fibrosis of multiple visceral organs 55-57. Our findings further extend this conserved profibrotic signaling axis to pathological cutaneous fibrosis and support gp130 as a potential therapeutic target for localized antifibrotic intervention.

Interestingly, our single-cell transcriptomic analysis revealed heterogeneous expression patterns of IL-11 signaling across fibroblast subsets.* IL11* was predominantly enriched in mesenchymal fibroblasts. Previous studies have demonstrated that this subset exhibits a secretory profibrotic phenotype and that conditioned medium derived from mesenchymal fibroblasts promotes collagen production in neighboring fibroblasts 43, supporting their paracrine profibrotic activity. Therefore, the enrichment of *IL11* in mesenchymal fibroblasts suggests that IL-11 may constitute one component of the profibrotic paracrine signals released by this fibroblast population. In contrast, *IL11RA* was broadly expressed across fibroblast subsets without clear subtype specificity, although relatively higher expression was observed in pro-inflammatory fibroblasts. This distribution suggests that multiple fibroblast subsets are potential effector cells of IL-11 signaling. The relatively higher expression of *IL11RA* in pro-inflammatory fibroblasts may indicate an increased responsiveness to IL-11 stimulation. However, the functional significance of this preferential expression remains to be further investigated.

Among the downstream signaling pathways associated with gp130 activation, we identified PI3K-AKT signaling as the most prominently suppressed pathway. The PI3K-AKT-mTOR axis has been extensively studied in cancer, where it regulates key cellular processes including cell metabolism, cytoskeletal dynamics, and protein synthesis 58, 59. Increasing evidence suggests that this pathway also contributes to fibrotic remodeling across multiple diseases, such as idiopathic pulmonary fibrosis 60, renal fibrosis 61, hepatic fibrosis 62, cardiac fibrosis 63, and systemic sclerosis 64. In fibroblasts, activation of PI3K-AKT-mTOR signaling has been linked to enhanced protein translation and matrix synthesis, indicating that this pathway may regulate the biosynthetic capacity of fibroblasts. Consistently, Bazedoxifene reduced phosphorylation of key components of this signaling cascade both *in vitro* and *in vivo*. Furthermore, mTOR activation was decreased in α-SMA⁺ myofibroblasts, suggesting that Bazedoxifene may restrain fibrotic remodeling partly by suppressing translational signaling in activated fibroblasts.

Our findings also highlight the potential for drug repurposing. Bazedoxifene is an FDA-approved selective estrogen receptor modulator with a well-established clinical safety profile. In this study, Bazedoxifene exhibited significant anti-fibrotic effects in both murine models, while no evident cytotoxicity was observed in dermal fibroblasts at the concentrations used. Interestingly, a recent study identified BAG2 and the downstream MEK signaling pathway as a potential therapeutic target in keloid disease and reported Bazedoxifene as a high-affinity ligand of BAG2 through large-scale compound screening 65, suggesting that Bazedoxifene may influence fibrotic processes through multiple intracellular signaling mechanisms. Taken together, these findings support the feasibility of repurposing Bazedoxifene as a pharmacological strategy for targeting fibrotic remodeling in pathological scarring. Given that pathological scarring is a localized skin disorder, localized drug delivery strategies may further enhance the therapeutic potential of Bazedoxifene while minimizing systemic exposure. For instance, transdermal delivery systems such as microneedle patches or hydrogel-based formulations may enable sustained local release of Bazedoxifene within scar tissue. Future studies evaluating local pharmacokinetics and tissue distribution will be important for optimizing its translational application.

Several limitations of this study should be acknowledged. First, although Bazedoxifene is known to disrupt IL-6/gp130 signaling, the causal relationship between gp130 inhibition and downstream PI3K-AKT-mTOR suppression was not directly validated through genetic rescue approaches. Second, while the two murine models provide useful platforms for studying fibrotic remodeling, these models cannot fully recapitulate the complex biomechanical and inflammatory microenvironment of human pathological scars. Finally, although our findings highlight the therapeutic potential of Bazedoxifene, further studies are required to characterize its local pharmacokinetic properties and tissue distribution to facilitate clinical translation.

## Conclusions

This study demonstrates that gp130 signaling is aberrantly activated in cutaneous fibrosis. Pharmacological inhibition of this pathway by Bazedoxifene suppresses fibroblast activation, including myofibroblast differentiation, fibroblast migration, and ECM production, primarily through attenuation of PI3K-AKT-mTOR signaling. These results position gp130 signaling as a key regulatory axis in cutaneous fibrosis and support the translational potential of Bazedoxifene as a therapeutic strategy for pathological cutaneous fibrosis.

## Supplementary Material

Supplementary methods, figures and tables.

## Figures and Tables

**Figure 1:**
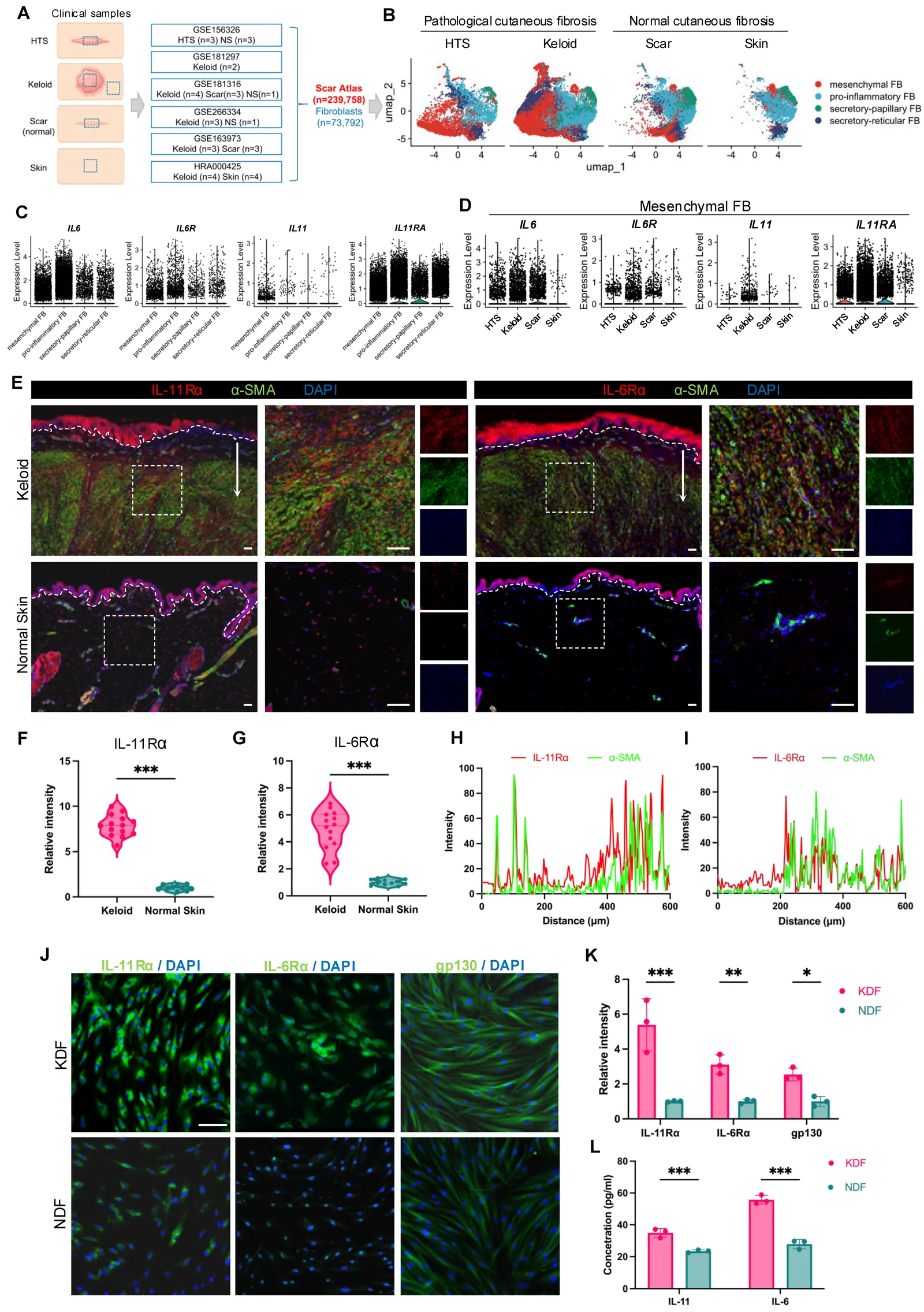
** Aberrant activation of gp130 signaling in pathological scars.** (A) Schematic overview of scRNA-seq data collection and integration workflow in this study. The datasets included keloid, hypertrophic scar (HTS), and normal controls (normal skin and normal scar). (B) UMAP of fibroblast subsets across different tissue types, including mesenchymal, pro-inflammatory, secretory-papillary, and secretory-reticular fibroblasts. (C) Violin plots showing the expression distribution of *IL6*, *IL6R*, *IL11*, and *IL11RA* across fibroblast subpopulations. (D) Violin plots showing the expression distribution of *IL6*, *IL6R*, *IL11*, and *IL11RA* in mesenchymal fibroblasts across HTS, keloid, scar, and normal skin. (E) Immunofluorescence staining of IL-11Rα or IL-6Rα (red), α-SMA (green) and DAPI (blue) in human keloid tissues and normal skin. Dashed boxes outline the fibrotic area. Arrows denote the direction along which the line-scan fluorescence intensity profile was generated to assess spatial distribution. Dashed lines show the epidermal-dermal barrier. Scale bars: 100 μm. (F) Quantification of IL-11Rα fluorescence intensity in dermal regions of keloid (n = 14) and normal skin (n = 10). (G) Quantification of IL-6Rα fluorescence intensity in dermal regions of keloid (n = 14) and normal skin (n = 10). (H) Line-scan fluorescence intensity profile showing the spatial distribution of IL-11Rα (red) relative to α-SMA (green) across the epidermis-dermis axis within a representative region of keloid tissue. (I) Line-scan fluorescence intensity profile showing the spatial distribution of IL-6Rα (red) relative to α-SMA (green) across the epidermis-dermis axis within a representative region of keloid tissue. (J) Representative immunofluorescence staining of IL-11Rα, IL-6Rα, and gp130 (green) in NDFs and KDFs. Scale bar: 100 μm. (K) Quantification of relative fluorescence intensity of IL-11Rα, IL-6Rα, and gp130 (green) in NDFs and KDFs (n = 3). (L) ELISA analysis of IL-6 and IL-11 levels in the culture supernatants of NDFs and KDFs (n = 3). Data are presented as mean ± SD. Statistical significance was determined using two-tailed Student's t-test. *P < 0.05*,* **P < 0.01*,* ***P < 0.001. HTS, hypertrophic scar; FB, fibroblast; NDF, normal dermal fibroblast; KDF, keloid-derived fibroblast.

**Figure 2:**
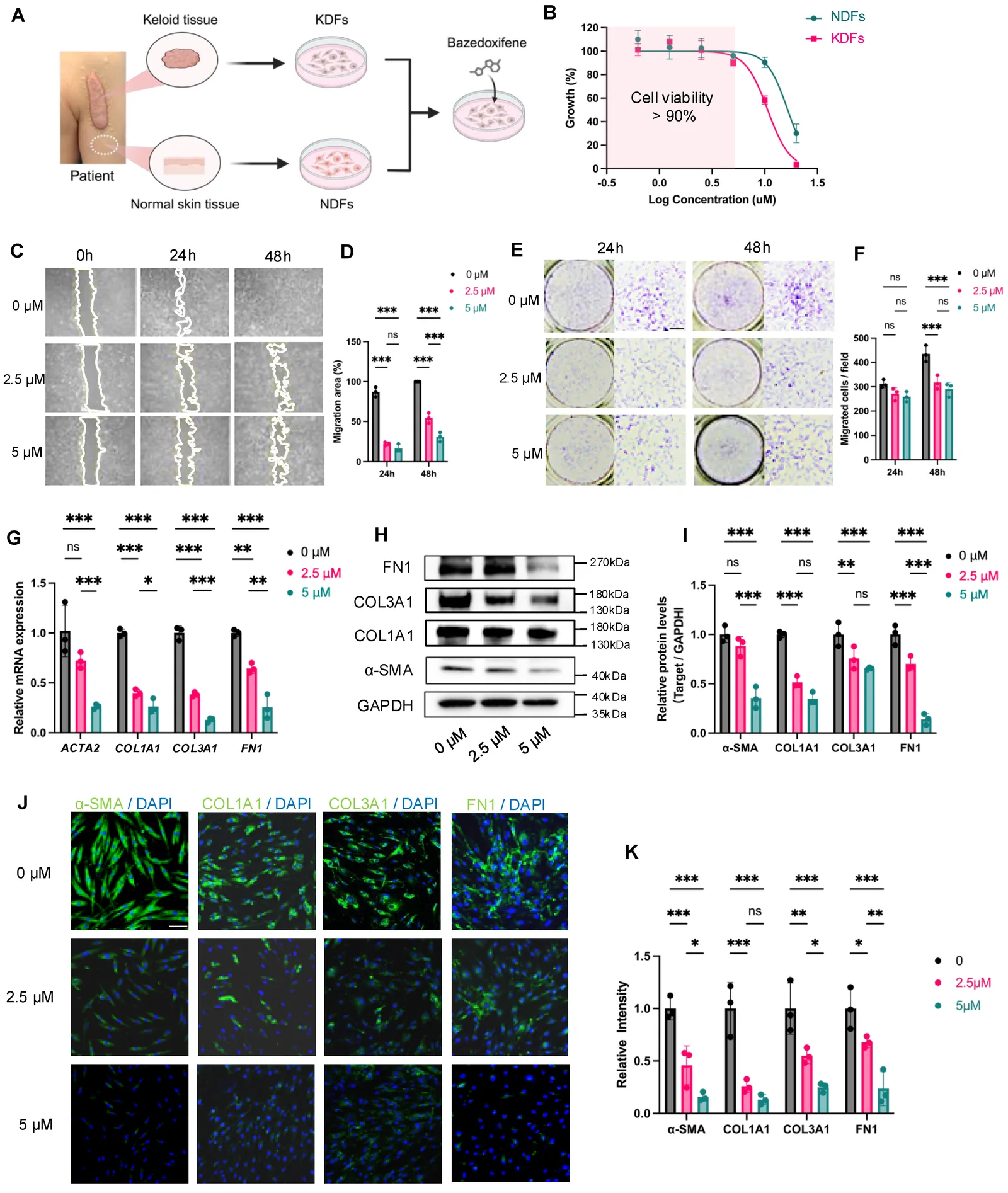
** Bazedoxifene inhibits fibroblast migration, myofibroblast differentiation, and ECM production in keloid-derived fibroblasts.** (A) Schematic illustration of the experimental design. Primary fibroblasts were isolated from keloid tissues and normal skin tissues obtained from patients and treated with Bazedoxifene for functional assays. (B) A CCK-8 assay was used to evaluate cell viability of NDFs and KDFs treated with increasing concentrations of Bazedoxifene for 48 h. The shaded area indicates concentrations at which cell viability remained above 90%. (C) Representative images of scratch wound healing assays showing KDF migration at 0 h, 24 h, and 48 h following treatment with Bazedoxifene (0, 2.5, and 5 μM). White lines indicate the wound boundaries. (D) Quantification of wound closure area in scratch assays. (E) Representative images of Transwell migration assays of fibroblasts treated with Bazedoxifene (0, 2.5, and 5 μM) for 24 h and 48 h. Scale bar: 500 μm. (F) Quantification of migrated cells per field in Transwell migration assays. (G) Relative mRNA expression levels of *ACTA2*, *COL1A1*, *COL3A1*, and *FN1* following Bazedoxifene treatment, determined by qRT-PCR. (H) Western blot analysis of FN1, COL3A1, COL1A1, and α-SMA protein expression relative to GAPDH in KDFs treated with Bazedoxifene. (I) Densitometric quantification of relative protein levels normalized to GAPDH. (J) Representative immunofluorescence staining of α-SMA, COL1A1, COL3A1, and FN1 (green) in KDFs treated with Bazedoxifene. (K) Quantification of relative fluorescence intensity of α-SMA, COL1A1, COL3A1, and FN1. Data are presented as mean ± SD (n = 3). Statistical significance was determined using one-way ANOVA followed by multiple comparisons test. *P < 0.05, **P < 0.01, ***P < 0.001. NDF, normal dermal fibroblast; KDF, keloid-derived fibroblast.

**Figure 3:**
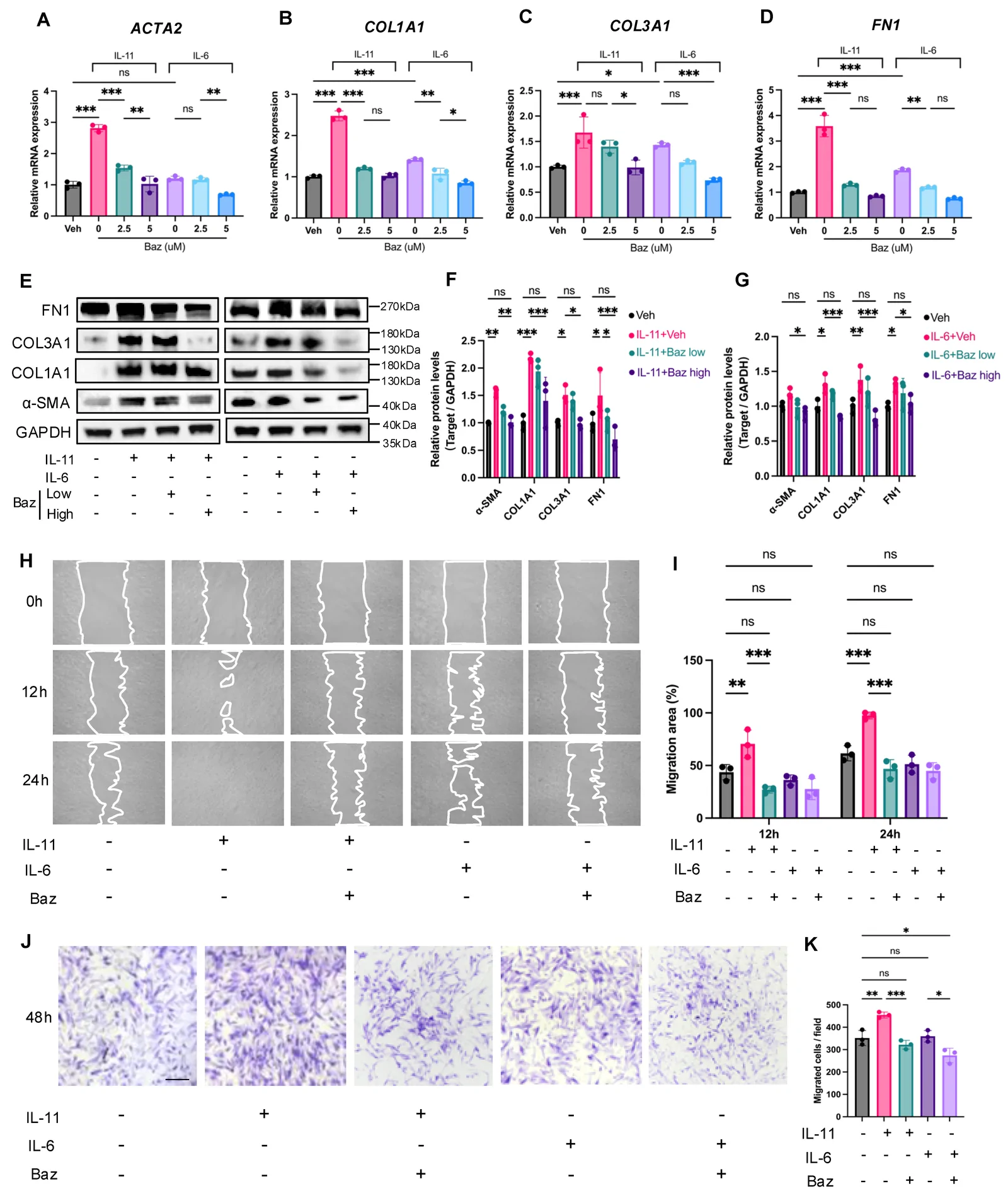
** Bazedoxifene attenuates IL-11/IL-6-induced fibroblast migration, myofibroblast differentiation, and ECM production.** (A-D) Relative mRNA expression levels of fibrotic genes *ACTA2* (A), *COL1A1* (B), *COL3A1* (C), and *FN1* (D) in NDFs under IL-11 or IL-6 stimulation with Bazedoxifene (0, 2.5, and 5 μM), as determined by qRT-PCR. (E) Western blot analysis of FN1, COL3A1, COL1A1, and α-SMA protein expression relative to GAPDH in NDFs under IL-11 or IL-6 stimulation with Bazedoxifene (Low, 2.5 μM; High, 5 μM). (F, G) Densitometric quantification of relative protein levels normalized to GAPDH under IL-11 stimulation (F) or IL-6 stimulation (G). (H) Representative images of scratch wound healing assays showing NDF migration at 0 h, 12 h, and 24 h under IL-11 or IL-6 stimulation with or without Bazedoxifene (5 μM). White lines indicate the wound boundaries. (I) Quantification of migration area in scratch wound healing assays. (J) Representative images of Transwell migration assays showing NDF migration at 48 h under IL-11 or IL-6 stimulation with or without Bazedoxifene (5 μM). (K) Quantification of migrated cells in Transwell migration assays. Data are presented as mean ± SD (n = 3). Statistical significance was determined using one-way ANOVA followed by multiple comparisons test. *P < 0.05*,* **P < 0.01*,* ***P < 0.001*.* NDF, normal dermal fibroblast; Baz, Bazedoxifene.

**Figure 4:**
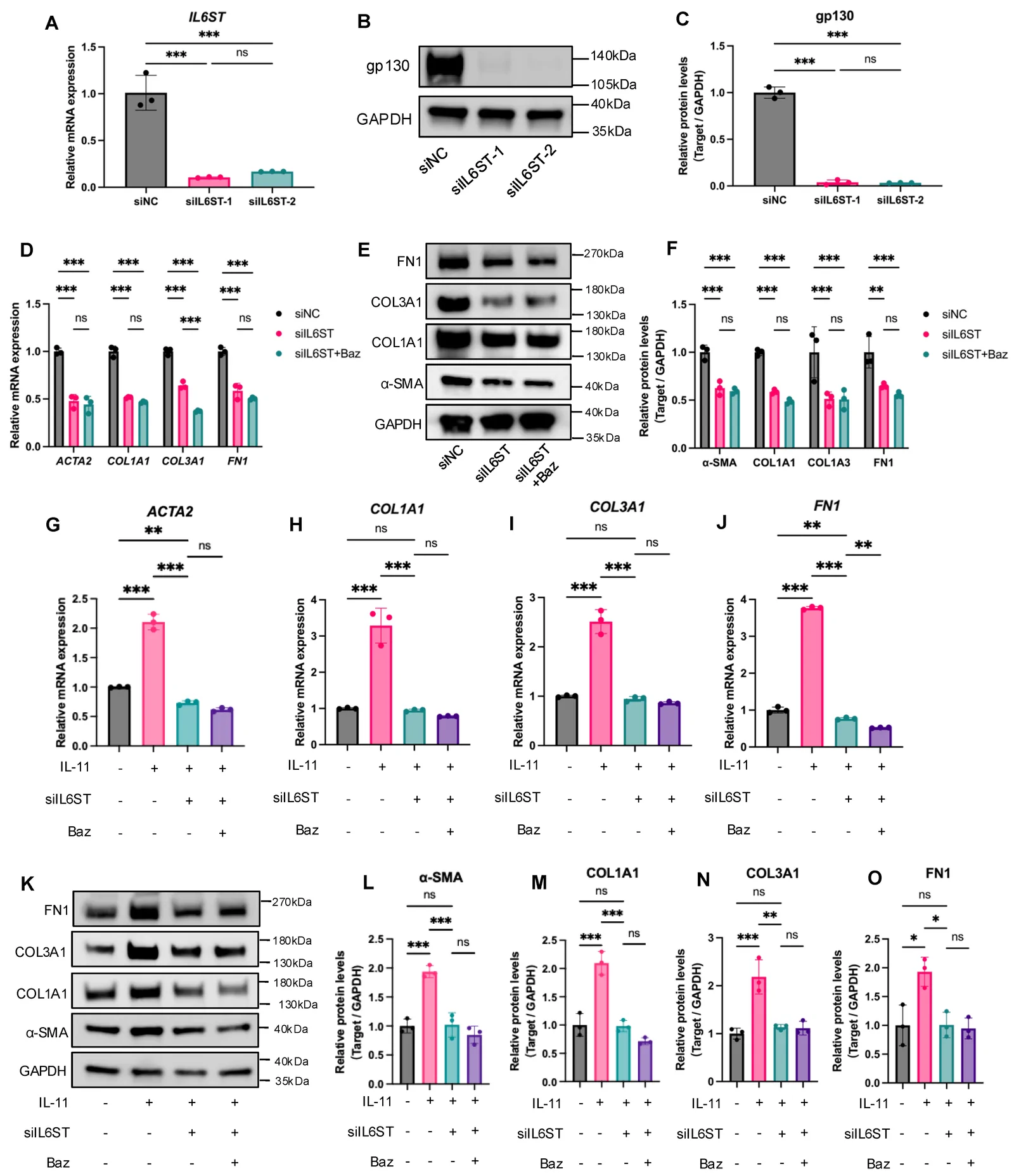
** Genetic silencing of IL6ST supports gp130 as the principal mediator of the antifibrotic effects of Bazedoxifene.** (A) Relative *IL6ST* mRNA expression in KDFs following transfection with two independent IL6ST siRNAs, as determined by qRT-PCR. (B) Western blot analysis of gp130 expression after *IL6ST* knockdown in KDFs. (C) Densitometric quantification of gp130 protein levels normalized to GAPDH. siIL6ST-1 was selected for subsequent experiments. (D) Relative mRNA expression of *ACTA2*, *COL1A1*, *COL3A1*, and FN1 in KDFs following *IL6ST* knockdown with or without Bazedoxifene treatment, as determined by qRT-PCR. (E) Western blot analysis of FN1, COL3A1, COL1A1, and α-SMA protein expression following* IL6ST* knockdown with or without Bazedoxifene treatment in KDFs. (F) Densitometric quantification of relative protein levels normalized to GAPDH. (G-J) Relative mRNA expression levels of *ACTA2*, *COL1A1*, *COL3A1*, and *FN1* in IL-11-stimulated NDFs following *IL6ST* knockdown with or without Bazedoxifene treatment, as determined by qRT-PCR. (K) Western blot analysis of FN1, COL3A1, COL1A1, and α-SMA protein expression in IL-11-stimulated NDFs following IL6ST knockdown with or without Bazedoxifene treatment. (L-O) Densitometric quantification of relative protein levels normalized to GAPDH. Data are presented as mean ± SD (n = 3). Statistical significance was determined using one-way ANOVA followed by multiple comparisons test. *P < 0.05, **P < 0.01, ***P < 0.001; ns, not significant*.*

**Figure 5:**
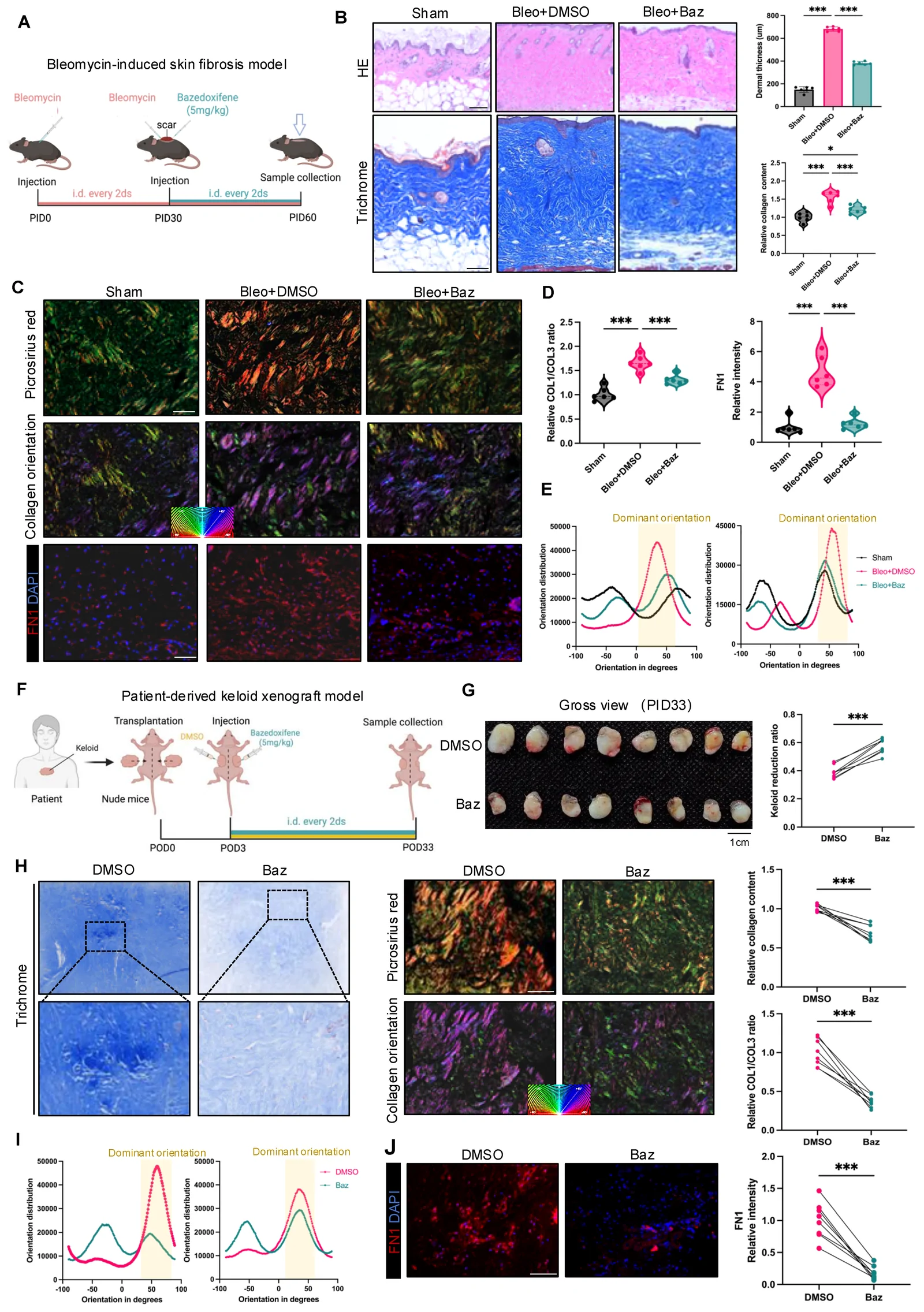
** Bazedoxifene attenuates dermal fibrosis *in vivo*.** (A) Study design of the bleomycin-induced skin fibrosis model and Bazedoxifene treatment regimen (n = 6 biologically independent mice per group). (B) Representative images of HE and Masson trichrome staining of skin tissues from Sham, Bleo+DMSO, and Bleo+Baz groups at post-injection day 60 (PID60). Scale bar: 100 μm. Quantification of dermal thickness and relative collagen content is shown on the right. Samples were collected from six mice per group, and data are presented as mean ± SD. Statistical significance was determined using one-way ANOVA followed by multiple comparisons test. *P < 0.05*,* **P < 0.01*,* ***P < 0.001. (C) Representative images of picrosirius red staining under polarized light (top), collagen orientation analysis (middle), and FN1 immunofluorescence staining (bottom) in skin tissues from the indicated groups. Collagen orientation analysis was conducted on picrosirius red-stained images using OrientationJ software. Scale bar: 100 μm. (D) Quantification of relative COL1/COL3 ratio (left) and relative FN1 density (right). Samples were collected from six mice per group, and data are presented as mean ± SD. Statistical significance was determined using one-way ANOVA followed by multiple comparisons test. *P < 0.05*,* **P < 0.01*,* ***P < 0.001*.* (E) Quantitative analysis of collagen fiber orientation distribution by OrientationJ software. Representative orientation distribution curves from two independent fields are shown. The Bleo+DMSO group exhibited a pronounced peak in orientation distribution, indicating increased collagen alignment along a dominant direction. Additional representative fields are presented in Figure S6A. (F) Study design of the patient-derived keloid xenograft model and Bazedoxifene treatment regimen (n = 8 biologically independent mice per group). (G) Gross view of excised keloid xenografts at postoperative day 33 (POD33) following treatment with DMSO or Bazedoxifene. Quantification of keloid reduction ratio is shown on the right. Data are presented as mean ± SD (n = 8 pairs of xenografts). Statistical significance was determined using paired two-tailed Student's t-test. *P < 0.05*,* **P < 0.01*,* ***P < 0.001. (H) Representative images of Masson trichrome staining (left), picrosirius red staining under polarized light (upper-middle), and collagen orientation analysis (lower middle) in xenograft tissues. Quantification of relative collagen content and relative COL1/COL3 ratio is shown on the right. Data are presented as mean ± SD (n = 8 pairs of xenografts). Statistical significance was determined using paired two-tailed Student's t-test. *P < 0.05*,* **P < 0.01*,* ***P < 0.001. (I) Quantitative analysis of collagen fiber orientation distribution in xenograft tissues using OrientationJ software. Representative orientation distribution curves from two independent fields are shown. The DMSO group exhibited a pronounced peak in orientation distribution, indicating increased collagen alignment, which was attenuated by Bazedoxifene treatment. Additional representative fields are shown in Figure S6B. (J) Representative FN1 immunofluorescence staining in xenograft tissues and quantification of relative FN1 density. Data are presented as mean ± SD (n = 8 pairs of xenografts). Statistical significance was determined using paired two-tailed Student's t-test. *P < 0.05*,* **P < 0.01*,* ***P < 0.001*.* Bleo, bleomycin; Baz, Bazedoxifene.

**Figure 6:**
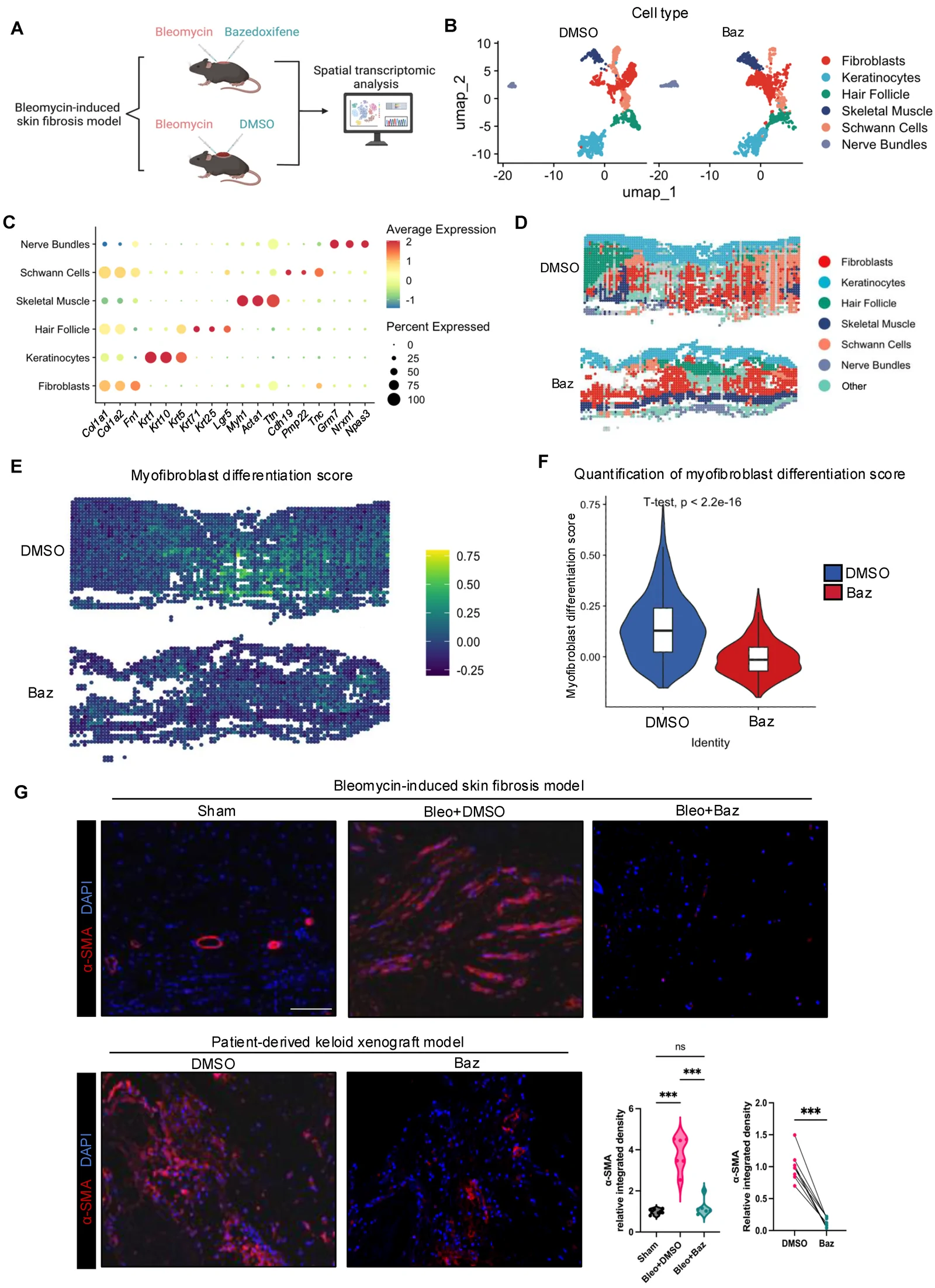
** Bazedoxifene suppresses myofibroblast differentiation *in vivo*.** (A) Scheme of the spatial transcriptomic analysis workflow. Skin tissues were collected from the bleomycin-induced fibrosis model. (B) Unsupervised clustering showing 6 cell types within the tissue. (C) Dot plot of representative marker gene expression for cell-type annotation of spatial clusters. (D) Cell-type annotation of spatial clusters, identifying fibroblasts, keratinocytes, hair follicle cells, skeletal muscle cells, Schwann cells, nerve bundles, and other cell types. (E) Spatial distribution of myofibroblast differentiation scores across tissue sections. (F) Quantitative comparison of myofibroblast differentiation scores between DMSO and Baz groups. Data are presented as mean ± SD. Statistical significance was determined using a two-tailed Student's t-test. (G) Representative immunofluorescence staining of α-SMA (red) and DAPI (blue) in dermal regions of skin tissues from the bleomycin-induced fibrosis model (upper) and patient-derived keloid xenograft model (lower left). Scale bars: 100 μm. Quantification of relative α-SMA-positive signal is shown on the lower right. Violin plots (left) represent the bleomycin-induced fibrosis model, and paired comparisons (right) represent the keloid xenograft model. Data are presented as mean ± SD. Statistical significance was determined using a two-tailed Student's t-test for the bleomycin-induced fibrosis model and a paired Student's t-test for the keloid xenograft model. *P < 0.05*,* **P < 0.01*,* ***P < 0.001; ns, not significant. Baz, Bazedoxifene; Bleo, bleomycin.

**Figure 7:**
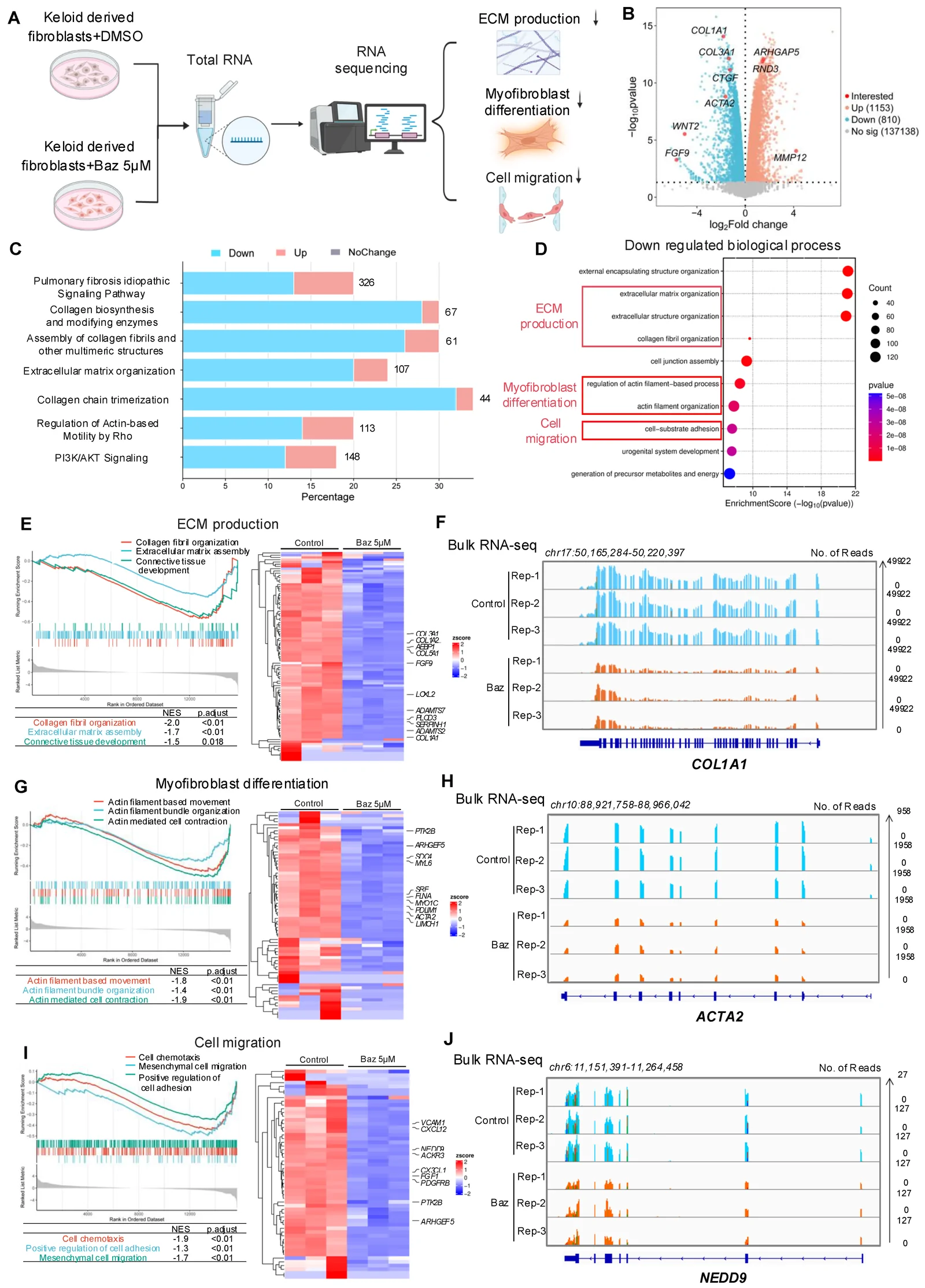
** Transcriptomic analysis reveals suppression of fibroblast activation-related signaling by Bazedoxifene.** (A) Schematic illustration of the RNA sequencing workflow. Keloid-derived fibroblasts were treated with Bazedoxifene (5 μM) or vehicle control followed by total RNA extraction and bulk RNA sequencing to investigate transcriptional changes. Transcriptomic analyses identified changes in pathways associated with ECM production, myofibroblast differentiation, and cell migration. (B) Volcano plot showing DEGs in Bazedoxifene-treated keloid fibroblasts compared with vehicle controls. Representative genes associated with fibrotic remodeling are marked. DEGs were identified according to the predefined cutoff criteria (|log2FC| ≥ 1, p < 0.05). (C) Ingenuity Pathway Analysis (IPA) of canonical signaling pathways enriched among DEGs following Bazedoxifene treatment. (D) Gene Ontology (GO) enrichment analysis of downregulated biological processes following Bazedoxifene treatment. (E, G, I) Gene set enrichment analysis (GSEA) demonstrating suppression of gene sets associated with ECM production (E), myofibroblast differentiation (G), and cell migration (I) in Bazedoxifene-treated fibroblasts compared with controls. Heatmaps show the expression patterns of representative genes within each gene set. (F, H, J) RNA-seq coverage tracks visualized using the Integrative Genomics Viewer (IGV) showing reduced transcriptional signals of representative genes involved in ECM production (*COL1A1*), myofibroblast differentiation (*ACTA2*), and cell migration (*NEDD9*). Data were generated from three biologically independent samples per group. DEG, differentially expressed gene.

**Figure 8:**
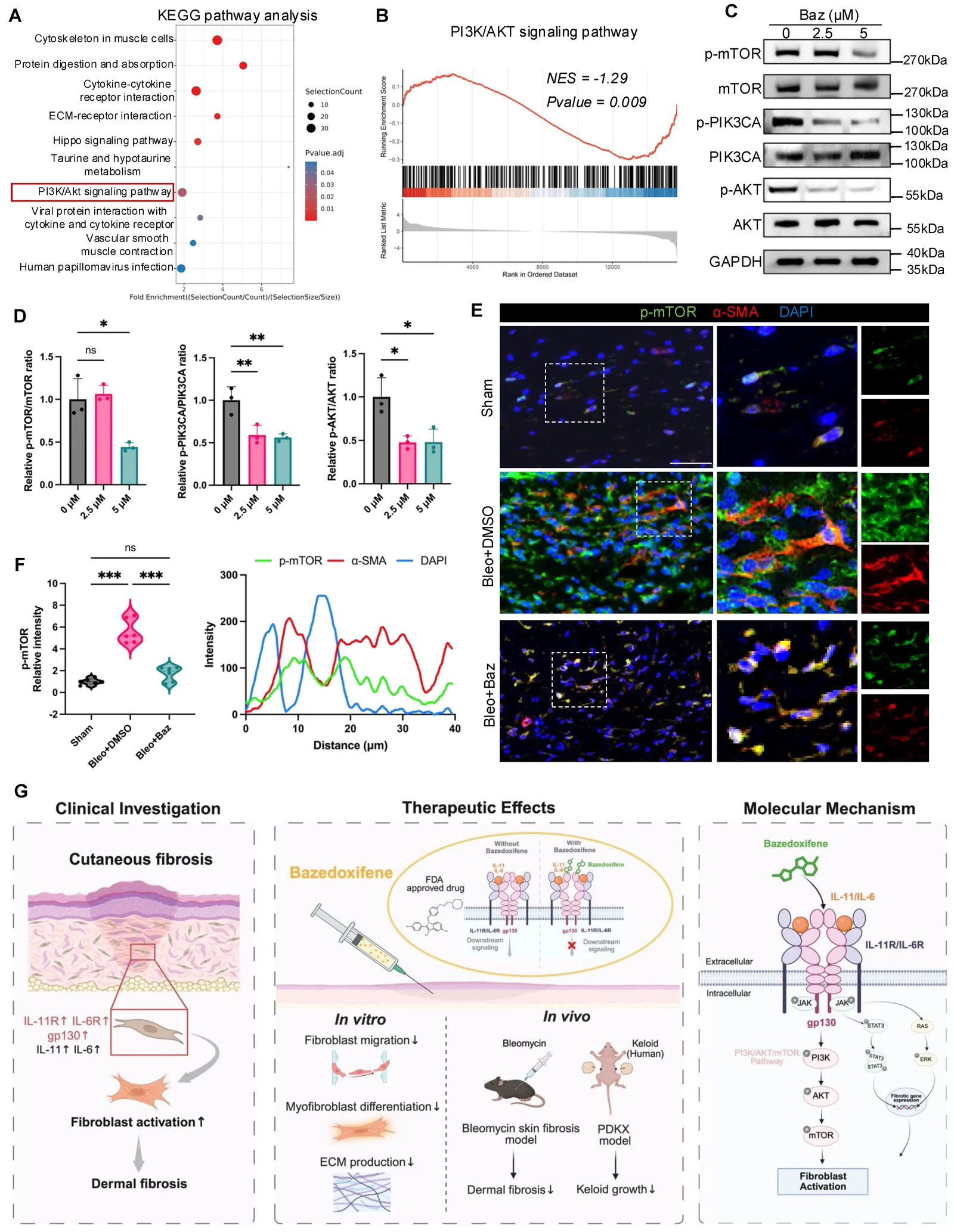
** Bazedoxifene Suppresses the PI3K-AKT-mTOR Pathway Downstream of the gp130 Axis.** (A) KEGG pathway enrichment analysis of DEGs in Bazedoxifene-treated keloid fibroblasts compared with vehicle controls. The PI3K/AKT signaling pathway was among the top significantly enriched pathways. (B) Gene set enrichment analysis (GSEA) demonstrating suppression of the PI3K/AKT signaling pathway following Bazedoxifene treatment. (C) Western blot analysis of p-mTOR, mTOR, p-PIK3CA, PIK3CA, p-AKT, and AKT protein expression relative to GAPDH in fibroblasts treated with Bazedoxifene (0, 2.5, and 5 μM). (D) Densitometric quantification of relative phosphorylation levels of mTOR, PIK3CA, and AKT normalized to their corresponding total proteins (n = 3). (E) Representative immunofluorescence staining of p-mTOR (green), α-SMA (red), and DAPI (blue) in skin tissues from the bleomycin-induced skin fibrosis model. Dashed boxes indicate the regions shown at higher magnification on the right. Scale bar: 100 μm. (F) Quantification of relative p-mTOR fluorescence intensity (n = 6, left) and line-scan fluorescence intensity profile (right) showing the spatial distribution of p-mTOR (green) relative to α-SMA (red). (G) Schematic Diagram illustrating that Bazedoxifene inhibits gp130-mediated signaling to fibroblast activation in cutaneous fibrosis, as validated by *in vitro* and *in vivo* models. Data are presented as mean ± SD. Statistical significance was determined using one-way ANOVA followed by multiple comparisons test. *P < 0.05*,* **P < 0.01*,* ***P < 0.001*.* DEG, differentially expressed gene. Bleo, bleomycin; Baz, Bazedoxifene.

## Data Availability

This study generated transcriptomic data. The bulk RNA-seq data are available from the Gene Expression Omnibus (GEO) repository GSE325896. The spatial transcriptomics data are available from the GEO repository GSE326315. This paper does not report original code. Any additional information required to reanalyze the data reported in this paper is available from the lead contact upon request.

## Abbreviations

ACTA2: alpha-smooth muscle actin
Baz: Bazedoxifene
CCK-8: Cell Counting Kit-8
COL1A1: collagen type I alpha 1 chain
COL3A1: collagen type III alpha 1 chain
CTGF: connective tissue growth factor
DAPI: 4′,6-diamidino-2-phenylindole
DAVID: Database for Annotation, Visualization and Integrated Discovery
DBiT-seq: deterministic barcoding in tissue sequencing
DEG: differentially expressed gene
DMEM: Dulbecco's modified Eagle's medium
DMSO: dimethyl sulfoxide
ECM: extracellular matrix
ELISA: enzyme-linked immunosorbent assay
FBS: fetal bovine serum
FDR: false discovery rate
FN1: fibronectin 1
GEO: Gene Expression Omnibus
GO: Gene Ontology
GO-BP: Gene Ontology Biological Process
GSA: Genome Sequence Archive
GSEA: Gene Set Enrichment Analysis
HE: hematoxylin and eosin
HTS: hypertrophic scar
IF: immunofluorescence
IGV: Integrative Genomics Viewer
IL-6: interleukin-6
IL-11: interleukin-11
IL6R: interleukin-6 receptor
IL11RA: interleukin-11 receptor alpha
IPA: Ingenuity Pathway Analysis
KDF: keloid-derived fibroblast
KEGG: Kyoto Encyclopedia of Genes and Genomes
NDF: normal dermal fibroblast
PBS: phosphate-buffered saline
PCA: principal component analysis
PDKX: patient-derived keloid xenograft
PI3K: phosphoinositide 3-kinase
PID: post-injection day
POD: postoperative day
qRT-PCR: quantitative reverse transcription polymerase chain reaction
RNA-seq: RNA sequencing
SD: standard deviation
scRNA-seq: single-cell RNA sequencing
STAT3: signal transducer and activator of transcription 3
TGF-β1: transforming growth factor beta 1
UMAP: Uniform Manifold Approximation and Projection
WB: Western blot
