# Supplementary material for: Bazedoxifene Targets gp130 Signaling to Suppress Fibroblast Activation in Pathological Cutaneous Fibrosis: Insights from Spatial Transcriptomics and scRNA-seq Data
Source: Int J Biol Sci. 2026 Aug 24;22(14):7491–511. doi: 10.7150/ijbs.137778 (PMC13617656; doi:10.7150/ijbs.137778)
Supplement: Supplementary file 1 [file ijbsv22p7491s1.pdf]

Supplementary methods

EdU Incorporation Assay

A total of 5,000–6,000 cells were seeded in 24-well plates and allowed to adhere overnight. Cells were then serum-starved for 12 h prior to treatment. After drug administration, cells were cultured for an additional 24 h, followed by evaluation of cell proliferation using an EdU Cell Proliferation Assay Kit (Invitrogen, Click-iT EdU Imaging Kit, C10337) according to the manufacturer's instructions. Briefly, cells were incubated with 50 μM EdU for 3.5 h before fixation, permeabilization, and EdU staining. Cell nuclei were subsequently stained with 4′,6-diamidino-2-phenylindole (DAPI; 1 μg/mL, Sigma–Aldrich, D9542) for 20 min. The proportion of EdU-positive cells was determined using a Zeiss LSM 710 laser scanning confocal microscope (Thornwood, NY, USA).

Apoptosis and Cell Cycle Assays

Cells were seeded in 6-well plates and allowed to adhere overnight, followed by serum starvation for 12 h prior to treatment. After treatment with the indicated agents for 24 h, apoptosis was assessed using a fluorescein isothiocyanate (FITC)-Annexin V apoptosis detection kit (BD Biosciences, San Diego, CA) according to the manufacturer's instructions. Briefly, cells were washed twice with cold PBS and stained with FITC-Annexin V and propidium iodide (PI) on ice for 5 min. For cell cycle analysis, cells were harvested after treatment, fixed in 75% cold ethanol at 4 °C for 2 h,

and then washed three times with PBS. Cells were subsequently stained with RNase A and PI (Cell Cycle Assay Kit, Dojindo, Japan) according to the manufacturer's instructions. All samples were analyzed using a BD LSRFortessa flow cytometer (BD Biosciences).

Supplementary figures

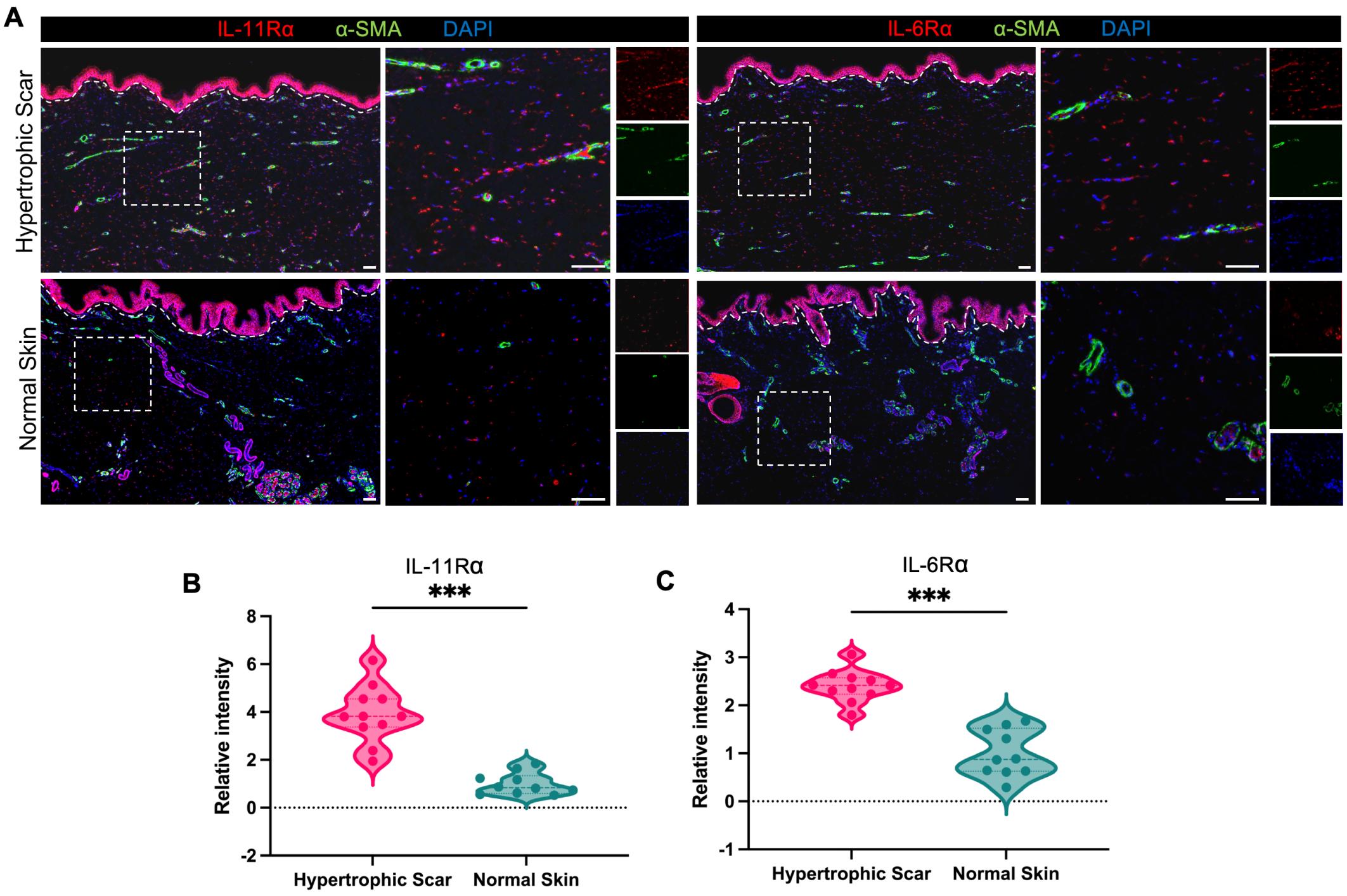

Figure S1. IL-11Rα and IL-6Rα are upregulated in hypertrophic scar tissues. (A) Immunofluorescence staining of IL-11Rα or IL-6Rα (red), α-SMA (green) and DAPI (blue) in hypertrophic scars (HTS) and normal skin. Dashed boxes outline the fibrotic area. Dashed lines show the epidermal-dermal barrier. Scale bars: 100 μm. (B) Quantification of IL-11Rα fluorescence intensity in dermal regions of HTS (n = 11) and normal skin (n = 10). (C) Quantification of IL-6Rα fluorescence intensity in dermal regions of HTS (n = 11) and normal skin (n = 10). Data are presented as mean ± SD. Statistical significance was determined using two-tailed Student's t-test. *$*P < 0.05$, $**P < 0.01$, $***P < 0.001$.*

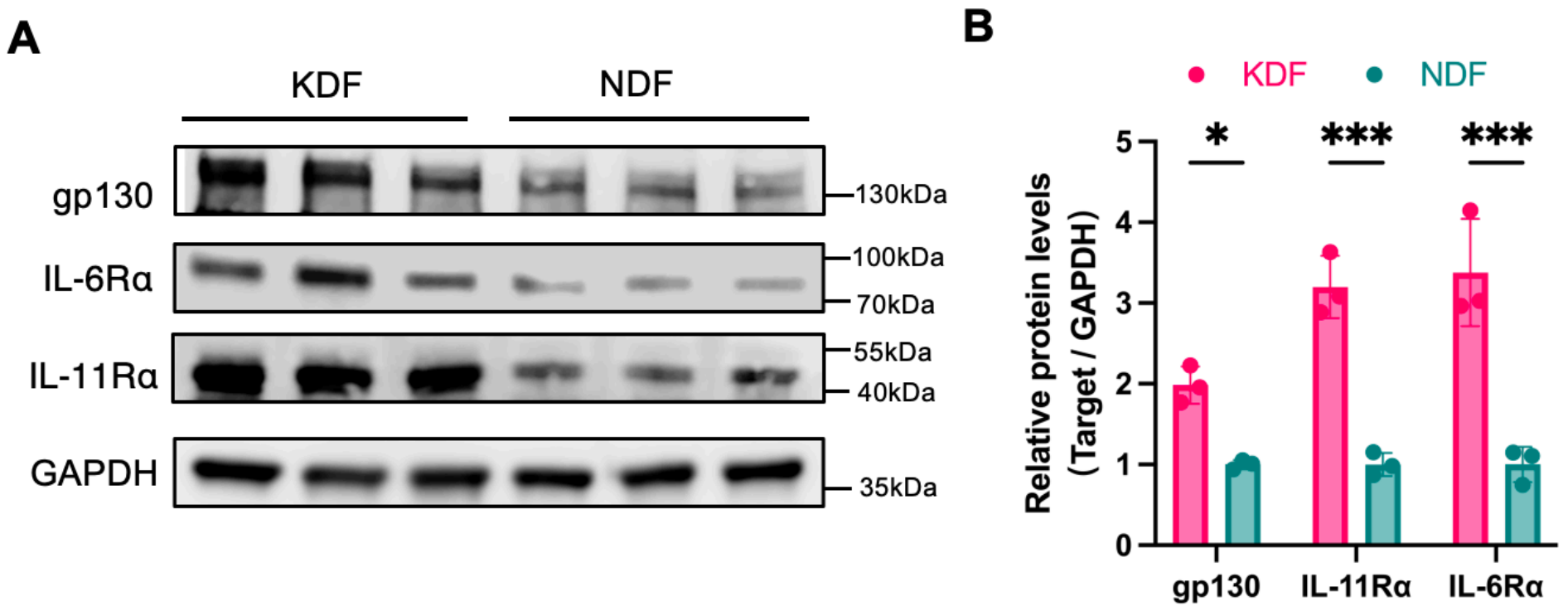

Figure S2. Protein levels of IL-11Rα, IL-6Rα, and gp130 in NDFs and KDFs. (A) Western blot analysis of IL-11Rα, IL-6Rα, and gp130 expression relative to GAPDH in NDFs and KDFs. (B) Densitometric quantification of relative protein levels normalized to GAPDH ($n = 3$). Data are presented as mean ± SD. Statistical significance was determined using two-tailed Student's t-test. *$P < 0.05$, **$P < 0.01$, ***$P < 0.001$. NDF, normal dermal fibroblast; KDF, keloid-derived fibroblast.

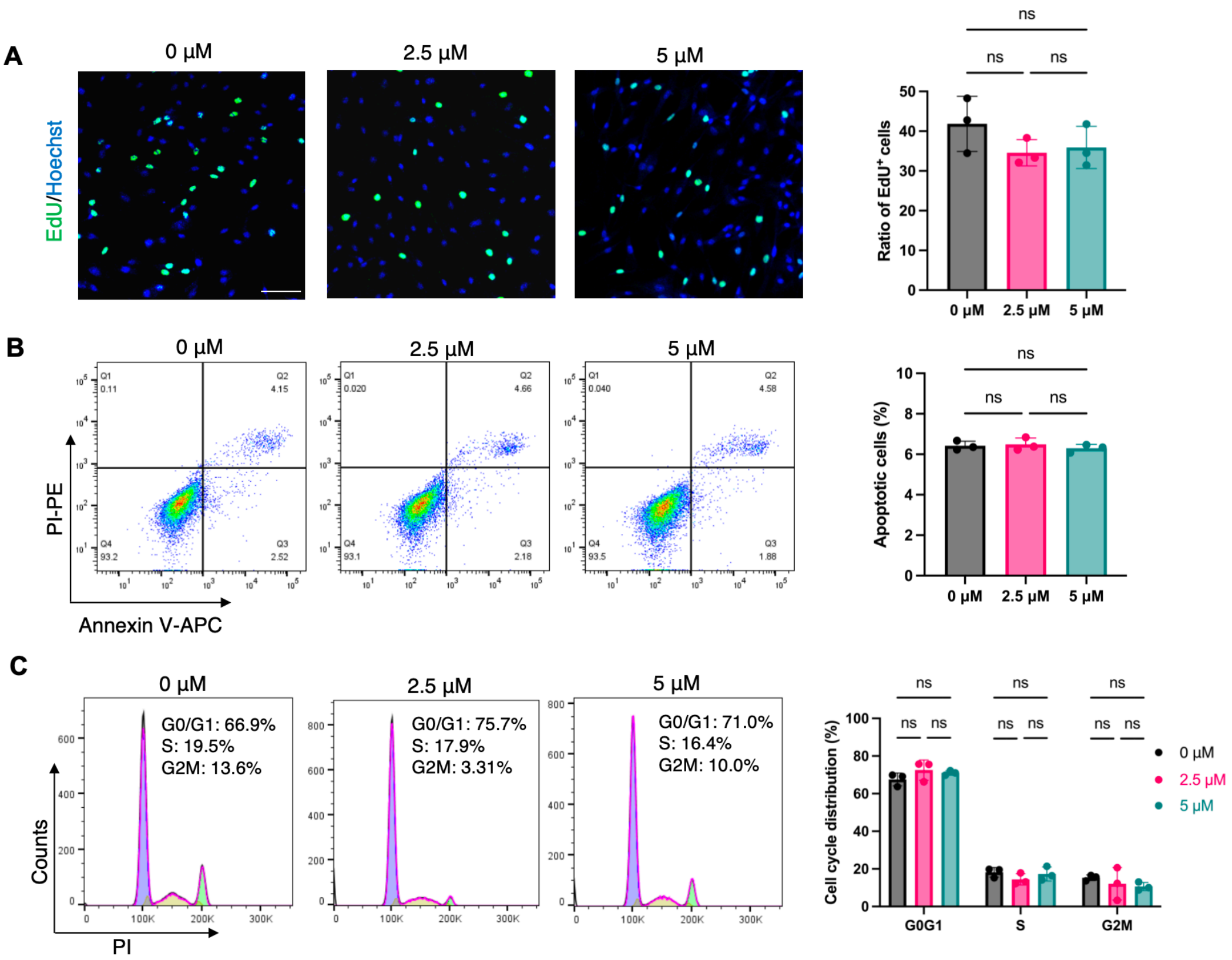

Figure S3. Bazedoxifene does not significantly affect proliferation, apoptosis, or cell cycle progression in keloid-derived fibroblasts. (A) Representative EdU (green) incorporation assay image of KDFs treated with Bazedoxifene (0, 2.5, and 5μM). Quantification of the percentage of EdU-positive cells is presented on the right. Scale bar: 100 μm. (B) Representative flow cytometry plots of Annexin V-APC/PI staining showing apoptotic cells following Bazedoxifene treatment (0, 2.5, and 5 μM). Quantification of apoptotic cell percentages is shown on the right. (C) Cell cycle analysis of fibroblasts treated with Bazedoxifene (0, 2.5, and 5 μM) assessed by PI staining and flow cytometry. Representative histograms and quantification of cell cycle distribution (G0/G1, S, and G2/M phases) are shown. Data are presented as mean ± SD (n = 3). Statistical significance was determined using one-way ANOVA followed by multiple comparisons test. ns, not significant. KDF, keloid-derived fibroblasts.

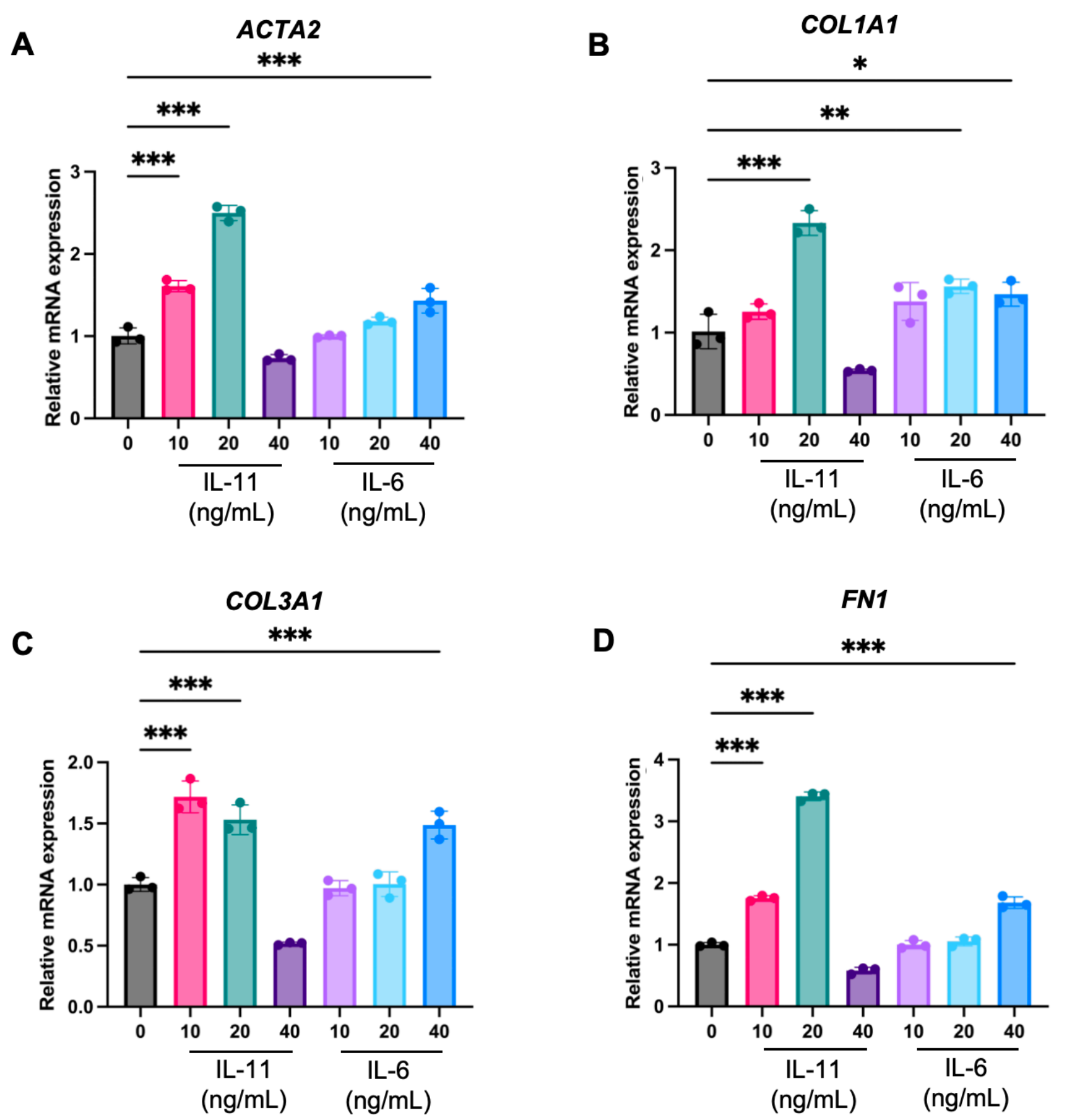

Figure S4. IL-11 and IL-6 stimulation induces fibrotic gene expression in normal dermal fibroblasts. (A-D) Relative mRNA expression levels of fibrotic genes *ACTA2* (A), *COL1A1* (B), *COL3A1* (C), and *FN1* (D) in normal dermal fibroblasts treated with increasing concentrations of IL-11 (10, 20, and 40 ng/mL) or IL-6 (10, 20, and 40 ng/mL), as determined by qRT-PCR. IL-11-induced fibrotic gene expression reached maximal levels at 20 ng/mL, whereas IL-6 showed the strongest effect at 40 ng/mL. Data are presented as mean±SD (n = 3). Statistical significance was determined using one-way ANOVA followed by multiple comparisons test. *$P < 0.05$, **$P < 0.01$, ***$P < 0.001$.

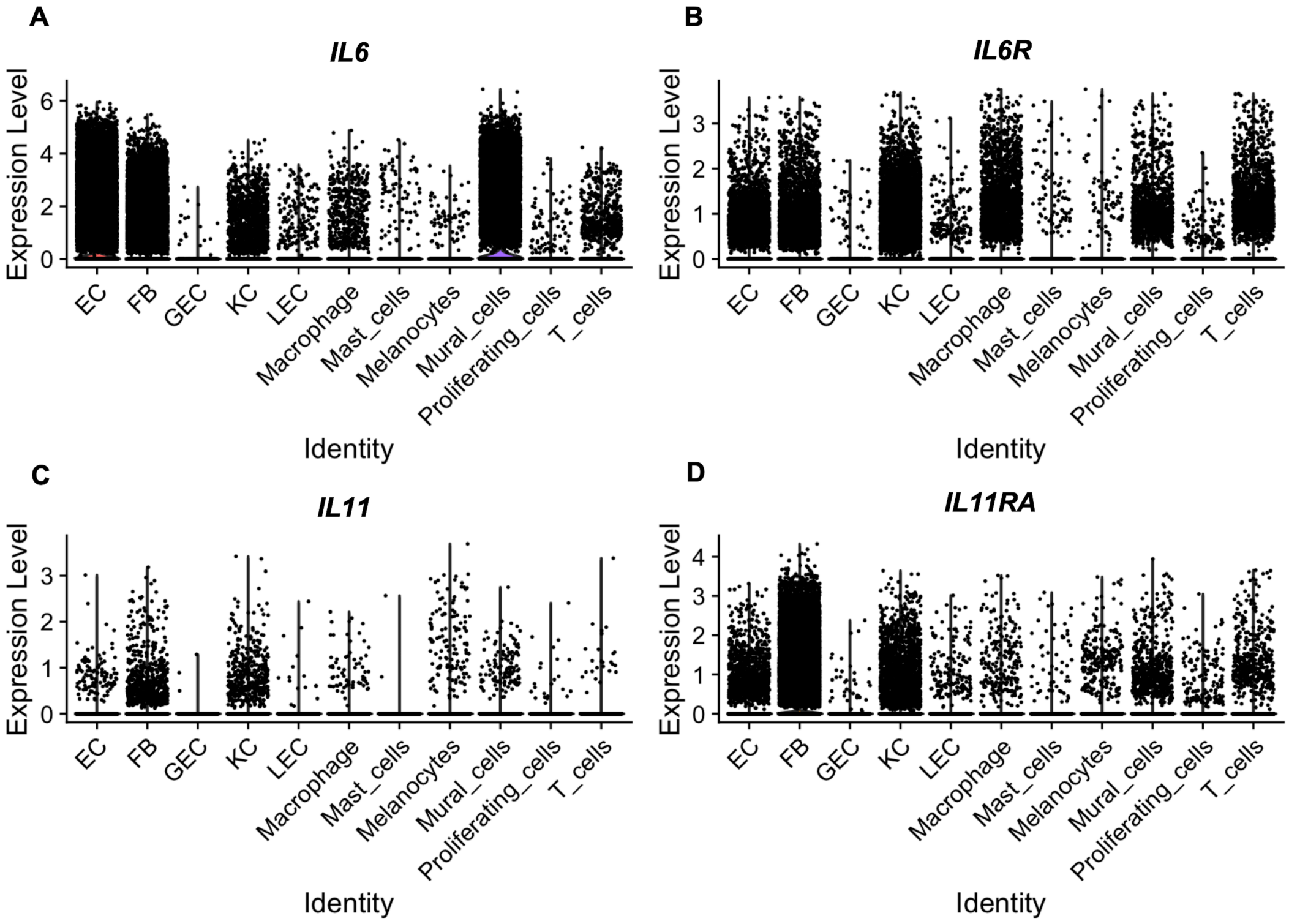

Figure S5. Expression of *IL6*, *IL6R*, *IL11*, and *IL11RA* across major cell populations in the integrated scRNA-seq dataset. (A–D) Violin plots showing the expression of *IL6* (A), *IL6R* (B), *IL11* (C), and *IL11RA* (D) across major cell populations, including endothelial cells (EC), fibroblasts (FB), glandular epithelial cells (GEC), keratinocytes (KC), lymphatic endothelial cells (LEC), macrophages, mast cells, melanocytes, mural cells, proliferating cells, and T cells.

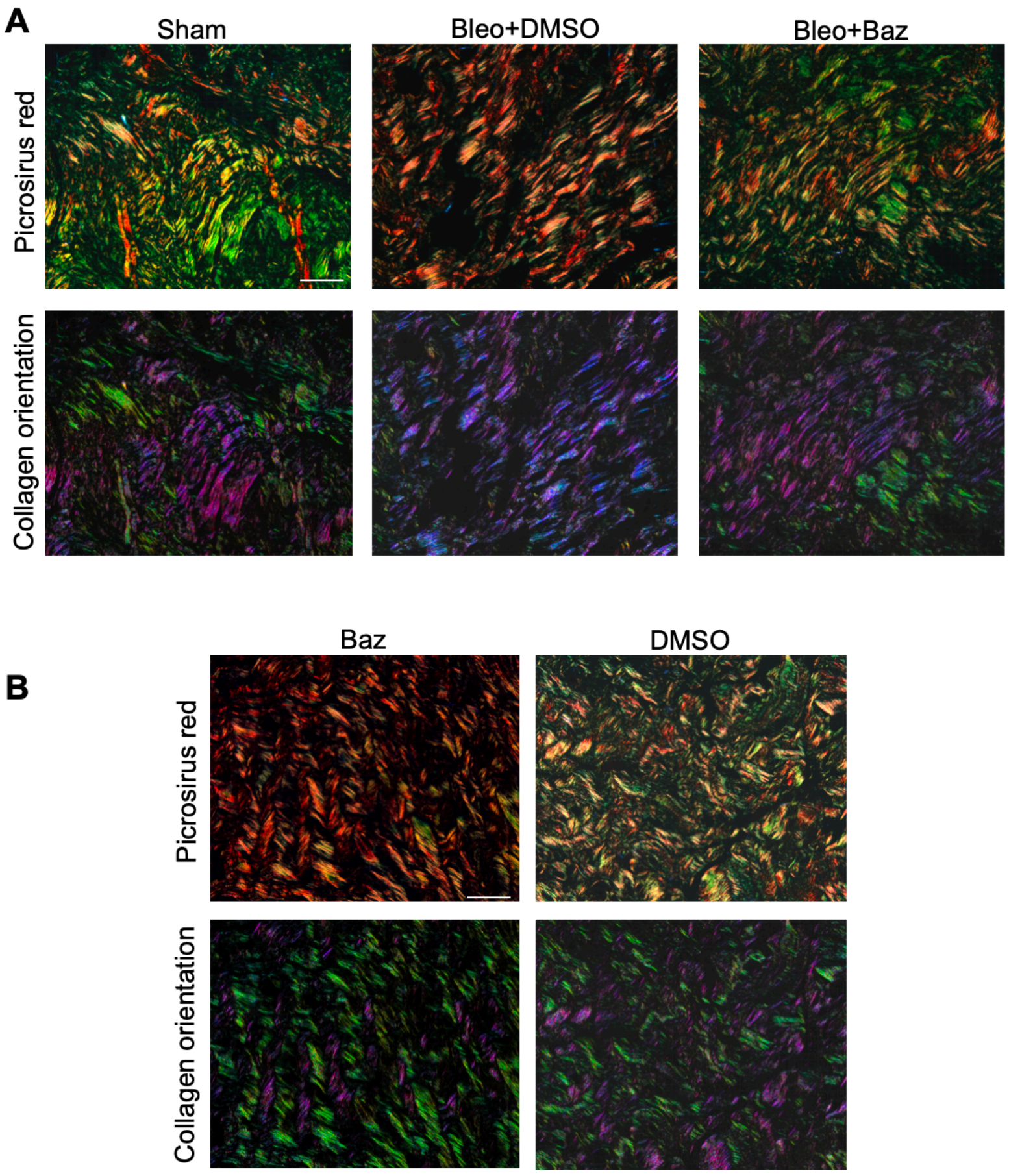

Figure S6. Additional representative fields showing collagen organization and orientation distribution *in vivo*. (A) Representative image of picrosirius red staining under polarized light (top) and corresponding collagen orientation analysis (bottom). (B) Representative images of picrosirius red staining (top) and collagen orientation analysis (bottom) from keloid xenograft tissues treated with DMSO or Bazedoxifene. Scale bar: 100 μm. Bleo, Bleomycin; Baz, Bazedoxifene.

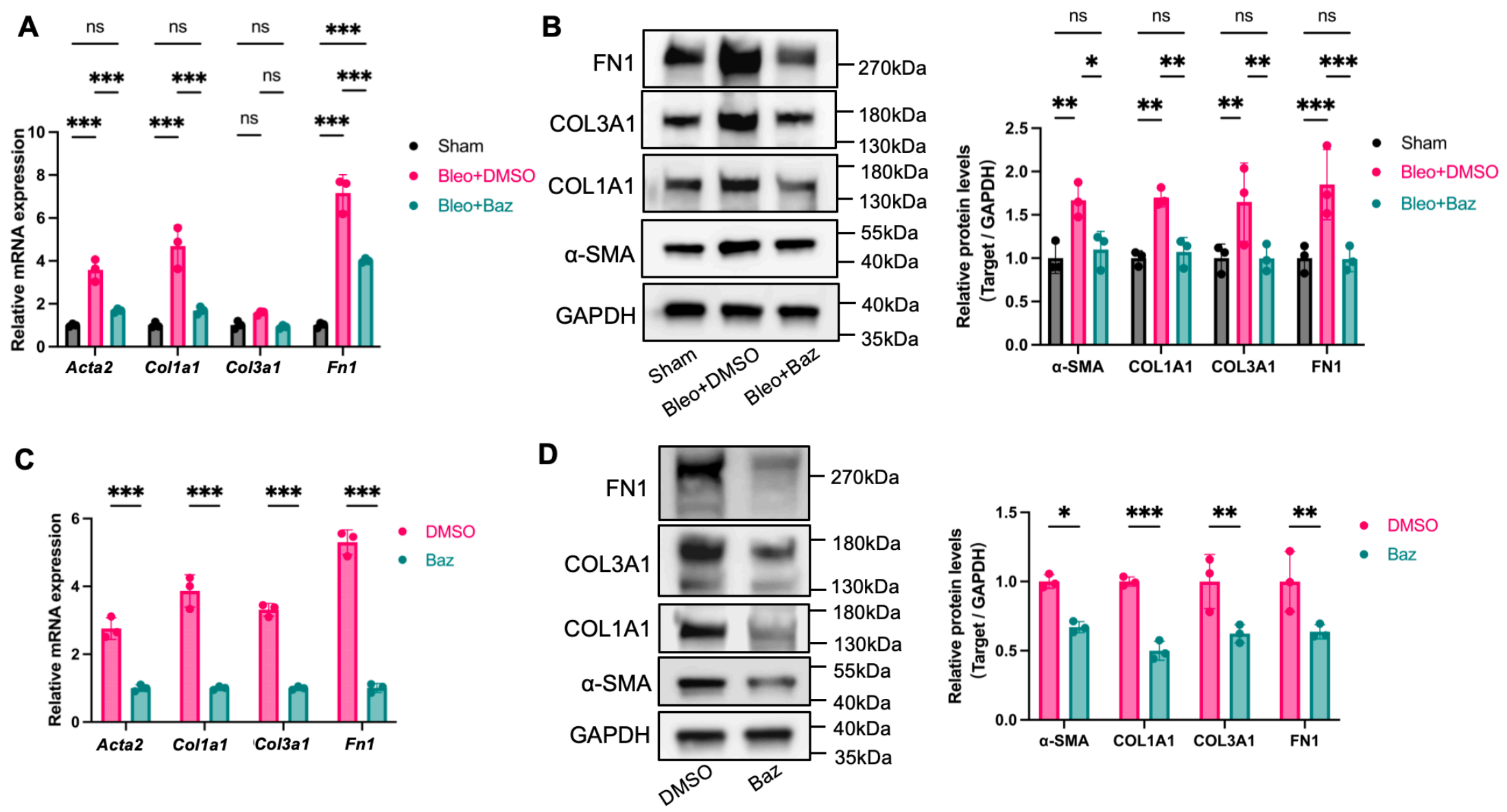

Figure S7. Bazedoxifene suppresses ECM production at both mRNA and protein levels *in vivo*. (A) Relative mRNA expression levels of *Acta2*, *Col1a1*, *Col3a1*, and *Fn1* in skin tissues from bleomycin-induced murine skin fibrosis model, as determined by qRT-PCR. Data are presented as mean±SD (n = 3). Statistical significance was determined using one-way ANOVA followed by multiple comparisons test. $^*P < 0.05$, $^{**}P < 0.01$, $^{***}P < 0.001$; ns, not significant. (B) Western blot analysis of FN1, COL3A1, COL1A1, and α-SMA protein expression relative to GAPDH in bleomycin-induced murine skin fibrosis model. Densitometric quantification of relative protein levels normalized to GAPDH is shown on the right. Data are presented as mean±SD (n = 3). Statistical significance was determined using one-way ANOVA followed by multiple comparisons test. $^*P < 0.05$, $^{**}P < 0.01$, $^{***}P < 0.001$; ns, not significant. (C) Relative mRNA expression levels of *Acta2*, *Col1a1*, *Col3a1*, and *Fn1* in skin tissues from patient-derived keloid xenograft model, as determined by qRT-PCR. Data are presented as mean±SD (n = 3). Statistical significance was determined using paired two-tailed Student's t-test. $^*P < 0.05$, $^{**}P < 0.01$, $^{***}P < 0.001$. (D) Western blot

analysis of FN1, COL3A1, COL1A1, and α-SMA protein expression relative to GAPDH in patient-derived keloid xenograft model. Densitometric quantification of relative protein levels normalized to GAPDH is shown on the right. Data are presented as mean±SD (n = 3). Statistical significance was determined using paired two-tailed Student's t-test. $^{*}P < 0.05$, $^{**}P < 0.01$, $^{***}P < 0.001$. Bleo, bleomycin; Baz, Bazedoxifene.

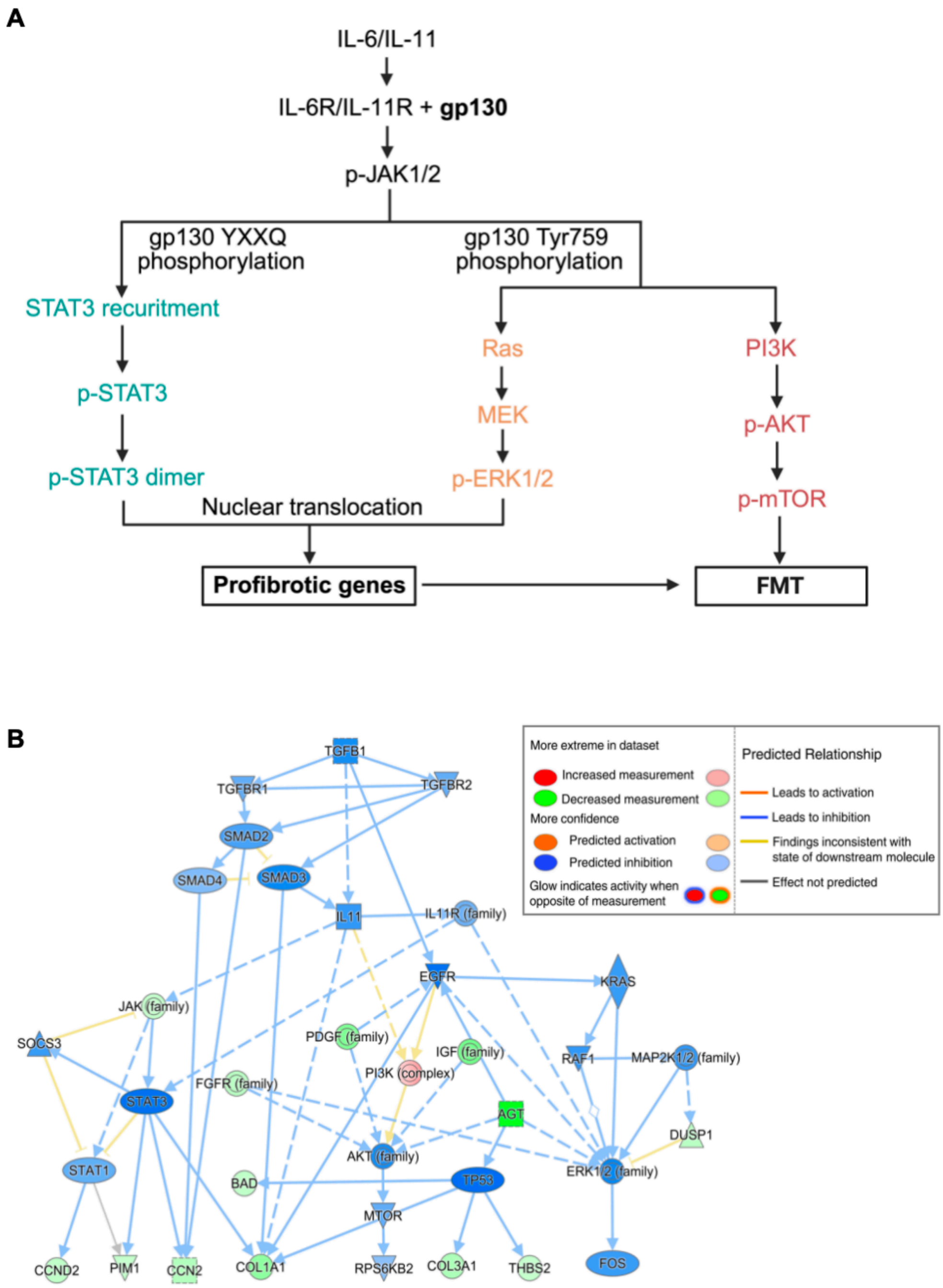

Figure S8. Canonical gp130 downstream signaling pathways and IPA-based network prediction analysis. (A) Schematic illustration of canonical downstream signaling pathways activated by IL-6/IL-11–gp130 signaling, including the JAK/STAT3, MAPK/ERK, and PI3K/AKT pathways. Activation of these pathways regulates fibrotic gene expression and fibroblast activation. (B) Ingenuity Pathway

Analysis (IPA)-based network prediction showing the global regulatory landscape of gp130-related signaling following Bazedoxifene treatment. The network highlights predicted inhibition of multiple downstream signaling nodes. Nodes represent molecules, and edges indicate predicted interactions. Color coding represents increased (red) or decreased (green) expression, and blue indicate predicted inhibition.

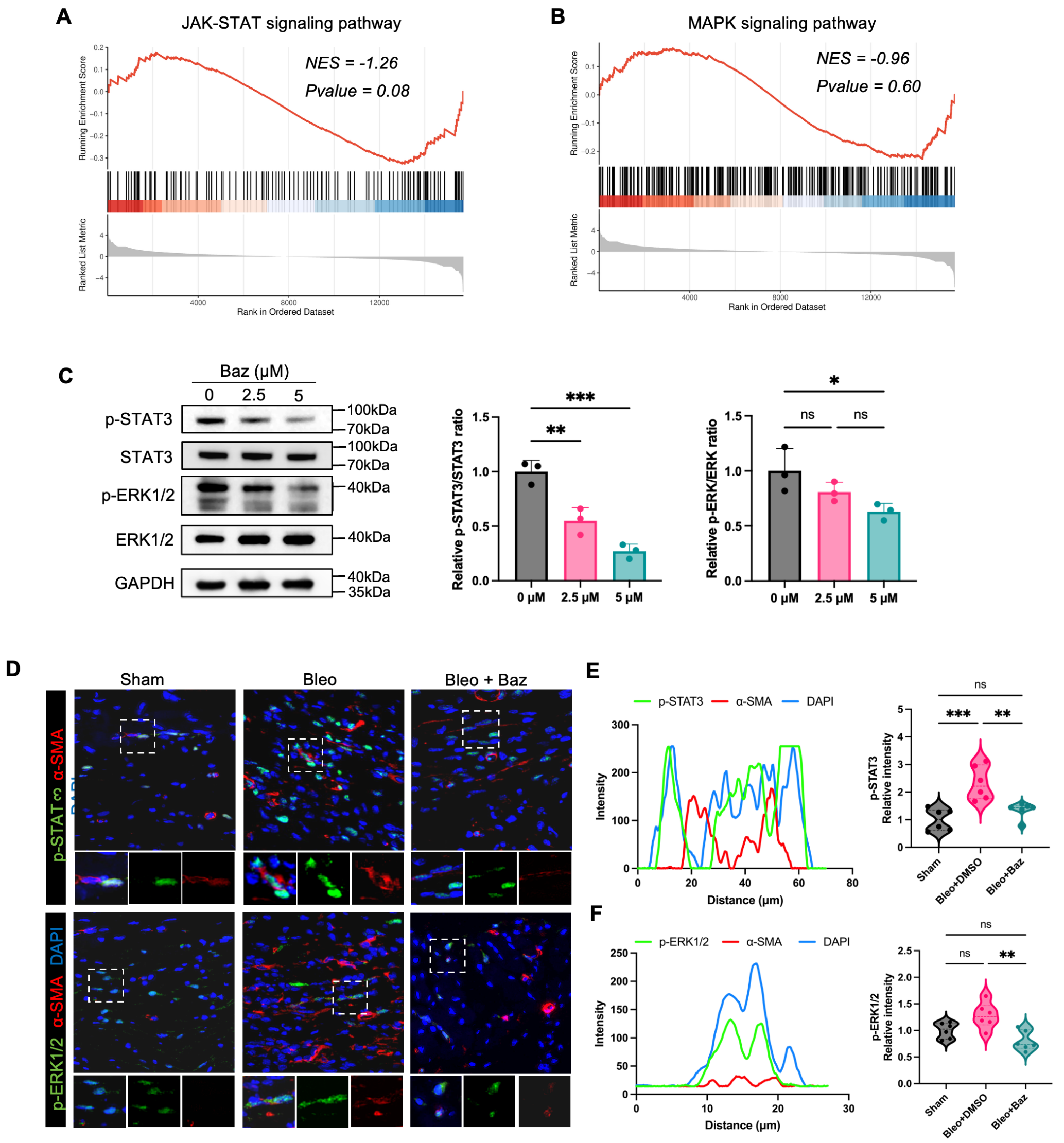

Figure S9. Suppression of STAT3 and MAPK/ERK signaling following Bazedoxifene treatment. (A, B) Gene set enrichment analysis (GSEA) showing the enrichment of the JAK-STAT (A) and MAPK/ERK (B) signaling pathways in fibroblasts treated with Bazedoxifene, both exhibiting a decreasing trend without statistical significance. (C) Western blot analysis of p-STAT3, STAT3, p-ERK1/2, and ERK1/2 in fibroblasts treated with Bazedoxifene (0, 2.5, and 5 μM). Quantification of relative phosphorylation levels (p-STAT3/STAT3 and p-ERK/ERK ratios) is shown on the right. (D) Representative immunofluorescence staining of p-STAT3 or p-ERK1/2

(green), α-SMA (red), and DAPI (blue) in skin tissues from bleomycin-induced murine skin fibrosis model. Dashed boxes indicate regions shown at higher magnification below. (E, F) Line-scan fluorescence intensity profiles of regions shown at higher magnification (left) and quantification (right) of relative p-STAT3 (E) and p-ERK1/2 (F) intensity in fibrotic regions. Data are presented as mean±SD. Statistical significance was determined using one-way ANOVA followed by multiple comparisons test. $*P < 0.05$, $**P < 0.01$, $***P < 0.001$; ns, not significant.

Supplementary tables

Table S1. Clinical information of skin and scar donors

Order	Sex	Age	Etiology	Body location	Scar age (month)	Experiments
Keloid1	Female	28	Surgery	Leg	9	IF
Keloid2	Male	45	Trauma	Arm	24	IF
Keloid3	Male	62	Burns	Back	48	IF
Keloid4	Female	35	Trauma	Earlobe	15	IF
Keloid5	Male	20	Surgery	Chest	26	Cell culture, IF, WB
Keloid6	Female	34	Burns	Chest	11	Cell culture, IF
Keloid7	Male	31	Burns	Neck	36	IF
Keloid8	Female	26	Trauma	Earlobe	22	IF
Keloid9	Female	39	Surgery	Abdomen	36	Cell culture, IF, WB
Keloid10	Male	36	Surgery	Chest	24	IF, PDKX
Keloid11	Female	23	Burns	Back	40	Cell culture, IF, WB
Keloid12	Male	57	Surgery	Chest	32	IF, PDKX
Keloid13	Female	28	Surgery	Arm	20	IF, PDKX
Keloid14	Female	46	Trauma	Face	8	IF, PDKX
Keloid15	Male	38	Trauma	Chest	28	PDKX
Keloid16	Female	31	Surgery	Abdomen	12	PDKX

Keloid17	Male	18	Trauma	Arm	24	PDKX
Keloid18	Male	40	Burns	Leg	50	PDKX
NS1	Female	28	-	Leg	-	IF
NS2	Female	34	-	Chest	-	IF
NS3	Male	31	-	Neck	-	IF
NS4	Male	36	-	Chest	-	Cell culture, IF
NS5	Female	23	-	Chest	-	Cell culture, IF, WB
NS6	Female	28	-	Arm	-	IF
NS7	Male	26	-	Arm	-	IF
NS8	Female	25	-	Back	-	IF
NS9	Female	26	-	Abdomen	-	IF
NS10	Male	36	-	Abdomen	-	Cell culture, IF, WB
NS11	Female	26	-	Back	-	Cell culture, WB
HTS1	Female	35	Surgery	Abdomen	12	IF
HTS2	Male	22	Trauma	Arm	18	IF
HTS3	Male	28	Burns	Chest	48	IF
HTS4	Female	40	Surgery	Neck	10	IF
HTS5	Female	28	Trauma	Leg	24	IF
HTS6	Male	32	Burns	Chest	60	IF
HTS7	Male	47	Trauma	Hand	15	IF
HTS8	Male	34	Surgery	Abdomen	20	IF
HTS9	Female	38	Burns	Chest	72	IF

HTS10	Male3	30	Trauma	Forehead	8	IF
HTS11	Female	25	Trauma	Leg	14	IF

IF, Immunofluorescence staining; WB, western blotting; PDKX, Patient-derived keloid xenograft.

Table S2. Primers used in experiments

Primer sequences for qRT–PCR			
ACTA2	Human	Forward	5′-AAAAGACAGCTACGTGGGTGA-3′
		Reverse	5′-GCCATGTTCTATCGGGTACTTC-3′
COL1A1	Human	Forward	5′-GTGCGATGACGTGATCTGTGA-3′
		Reverse	5′-CGGTGGTTTCTTGGTCGGT-3′
COL3A1	Human	Forward	5′-TTGAAGGAGGATGTTCCCATCT-3′
		Reverse	5′-ACAGACACATATTTGGCATGGTT-3′
FN1	Human	Forward	5′-CGGTGGCTGTCAGTCAAAG-3′
		Reverse	5′-AAACCTCGGCTTCCTCCATAA-3′
GAPDH	Human	Forward	5′-ATGTTGCAACCGGGAAGGA-3′
		Reverse	5′-CAGGAGCGCAGGGTTAGTC-3′
Acta2	Mouse	Forward	5′-GAGGCACCACTGAACCCTAA-3′
		Reverse	5′-TACATGGCGGGGACATTGAA-3′
Col1a1	Mouse	Forward	5′-TTCAGGGAATGCCTGGTGAA-3′
		Reverse	5′-ACCTTTGGGACCAGCATCA-3′
Col3a1	Mouse	Forward	5′-TGCTGGAAAGAATGGGGAGAC-3′
		Reverse	5′-GGTCCAGAATCTCCCTTGTCAC-3′
Fn1	Mouse	Forward	5′-CGTCATTGCCCTGAAGAACA-3′
		Reverse	5′-AAGGGTAACCAGTTGGGGAA-3′
Gapdh	Mouse	Forward	5′-ATGGGAAGCTTGTCATCAACG-3′
		Reverse	5′-GGCAGTGATGGCATGGACTG -3′

Table S3. Primary antibodies used in experiments

Antibodies	Source	Identifier	Dilution
Anti-IL-11R alpha	Proteintech	10264-1-AP	IF: 1:200; WB: 1:1000;
Anti-IL-6R alpha	Abcam	ab271042	IF: 1:50; WB: 1:1000
Anti-gp130/IL6ST	Proteintech	67766-1-Ig	IF: 1:200; WB: 1:1000
Anti-GAPDH	Proteintech	10494-1-AP	WB: 1:5000
Anti-Fibronectin 1	Proteintech	15613-1-AP	IF: 1:500; WB: 1:1000
Anti-Collagen III	Proteintech	22734-1-AP	IF: 1:500; WB: 1:1000
Anti-Collagen I	Proteintech	14695-1-AP	IF: 1:500; WB: 1:1000
Anti-αSMA	Abcam	ab32575	IF: 1:500; WB: 1:1000
Anti-Phospho-mTOR (Ser2448)	Proteintech	67778-1-Ig	IF: 1:50; WB: 1:1000
Anti-mTOR	Proteintech	66888-1-Ig	WB: 1:1000
Anti-Phospho-PIK3CA(Tyr317)	Affinity	AF4369	WB: 1:1000
Anti-PIK3CA	Abcam	ab40776	WB: 1:1000
Anti-Phospho-AKT(Ser473)	Abclonal	AP0637	WB: 1:1000

Anti-AKT	Proteintech	10176-2-AP	WB: 1:1000
Anti-Phospho-Stat3 (Tyr705)	Cell Signaling	#9145	IF: 1:100; WB: 1:1000
Anti-Stat3 (Tyr705)	Cell Signaling	#9139	WB: 1:1000
Anti-Phospho-ERK1/2 (Thr202/Tyr204)	Proteintech	28733-1-AP	IF: 1:100; WB: 1:1000
Anti-ERK1/2	Proteintech	11257-1-AP	WB: 1:1000
Anti-mouse IgG (H+L), F(ab')2 Fragment(Alexa Fluor ® 594 Conjugate)	Cell Signaling	#8890	IF: 1:400
Anti-rabbit IgG (H+L), F(ab')2 Fragment(Alexa Fluor ® 594 Conjugate)	Cell Signaling	#8889S	IF: 1:400
Anti-mouse IgG (H+L), F(ab')2 Fragment(Alexa Fluor ® 488 Conjugate)	Cell Signaling	#4408S	IF: 1:400
Anti-rabbit IgG (H+L), F(ab')2 Fragment(Alexa Fluor ® 488 Conjugate)	Cell Signaling	#4412	IF: 1:400

Table S4. siRNA sequences used for *IL6ST* knockdown.

Name	Sequence (5’–3’)
siIL6ST-1	AGGACCUACUGUUCGGACAAATT
	UUUGUCCGAACAGUAGGUCCUTT
siIL6ST-2	CAGAACUGCAGUCAGCAUGAATT
	UUCAUGCUGACUGCAGUUCUGTT
